# Rapid benefits in older age from transition to whole food diet regardless of protein source or fat to carbohydrate ratio: Arandomised control trial

**DOI:** 10.1111/acel.14276

**Published:** 2024-07-16

**Authors:** Rosilene V. Ribeiro, Alistair M. Senior, Stephen J. Simpson, Jian Tan, David Raubenheimer, David Le Couteur, Laurence Macia, Andrew Holmes, Joerg Eberhard, John O'Sullivan, Yen Chin Koay, Alisar Kanjrawi, Jean Yang, Taiyun Kim, Alison Gosby

**Affiliations:** ^1^ School of Life and Environmental Sciences University of Sydney Sydney New South Wales Australia; ^2^ Charles Perkins Centre University of Sydney Sydney New South Wales Australia; ^3^ Chronic Disease Theme, School of Medical Sciences, Faculty of Medicine and Health University of Sydney Sydney New South Wales Australia; ^4^ Centre for Education and Research on Ageing and Ageing and Alzheimers Institute, Concord Hospital University of Sydney Sydney New South Wales Australia; ^5^ ANZAC Research Institute University of Sydney, Concord Hospital Sydney New South Wales Australia; ^6^ Sydney Cytometry University of Sydney Sydney New South Wales Australia; ^7^ The University of Sydney School of Dentistry, Faculty of Medicine and Health University of Sydney Sydney New South Wales Australia; ^8^ Cardiometabolic Medicine, School of Medical Sciences, Faculty of Medicine and Health The University of Sydney Sydney New South Wales Australia; ^9^ Department of Cardiology, Royal Prince Alfred Hospital Camperdown New South Wales Australia; ^10^ School of Mathematics and Statistics University of Sydney Sydney New South Wales Australia

**Keywords:** aged, appetite, dietary carbohydrate, dietary fats, plant proteins

## Abstract

Plant‐based diets reduces the risk of chronic conditions. The interaction between protein source and other macronutrients—fat (F) and carbohydrate (C)—has yet to be investigated. The aim was to assess the main and interactive effects of protein‐source (plant vs. animal) and F:C (high or low) and the transition from an Australian diet to a whole food diet on various health markers in older individuals. This single‐blinded, parallel, randomised experimental trial used a 2 × 2 factorial design to compare pro‐vegetarian (70:30 plant to animal) versus omnivorous (50:50 plant to animal) diets at 14% protein and varying fat‐to‐carbohydrate ratios (high fat ~40% vs. low fat ~30%) over 4 weeks. Study foods were provided, alcohol consumption was discouraged, and dietary intake was determined through food records. Analysis included both RCT and observational data. Changes in appetite, palatability of diets, and dietary intake were assessed. Body composition, muscle strength, function, gut microbiome, and cardiometabolic health parameters were measured. Data from 113 (of the 128 randomised) individuals aged 65–75 years were analysed. Pro‐vegetarian diets reduced diastolic blood pressure, total cholesterol and glucose levels. Moreover, the overall sample exhibited increased short‐chain fatty acids and FGF21 levels, as well as improvements in body composition, function, and cardio‐metabolic parameters irrespective of dietary treatment. Transitioning to a diet rich in fruit, vegetables, fibre, and moderate protein was associated with improved health markers in older age, with added benefits from pro‐vegetarian diets. Further research on long‐term effects is needed.

## Graphical abstract

Rapid benefits in older age from transition to whole food diet regardless of protein source or fat to carbohydrate ratio: a randomised control trial. Individuals aged 65‐75 years were randomised to one of four diets and provided with all their food for 4 weeks. The overall sample exhibited increased acetate levels, as well as improvements in body composition, physical strength, and cardiometabolic parameters, irrespective of dietary treatment. Pro‐vegetarian diets reduce diastolic blood pressure, and pro‐vegetarian, high carbohydrate diet improved gut microbiome diversity.

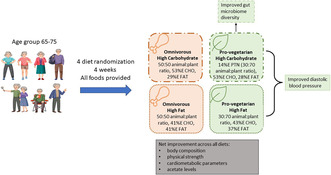

**Abbreviations**
AIC: Akaike information criterion
ALDEx2: analysis of differential abundance taking sample variation into account
ANOVA: analysis of variance
ASV: amplicon sequence variants
AUSNUT: Australian Food, Supplement and Nutrient Database
BCAA: branched‐chain amino acids
BL: baseline assessment
BMI: body mass index
C: carbohydrate
CLR: centre log‐ratio
DBP: diastolic blood pressure
F: fat
F:C: fat‐to‐carbohydrate ratio
FGF‐21: fibroblast growth factor 21
FN: final assessment after four‐week intervention
GAMM: generalised additive mixed model
GLMM: generalised linear mixed model
HDLc: high‐density lipoprotein cholesterol
HOMA‐IR: homeostatic model assessment for insulin resistance
ITT: intention to treat
LC‐MS/MS: liquid chromatography–tandem mass spectrometry
LDLc: low‐density lipoprotein cholesterol
MJ: megajoule
OHC: omnivorous, high carbohydrate
OHF: omnivorous, high fat
P:C: protein to carbohydrate ratio
PCR: polymerase chain reaction
PDF: plant‐derived foods
PERMANOVA: permutational multivariate analysis of variance
RCT: randomised controlled trial
RDP: ribosomal database project
SBP: systolic blood pressure
SCFA: short‐chain fatty acids
VHC: semi‐vegetarian, high carbohydrate
VHF: semi‐vegetarian, high fat
WFR: weighed food records

## 1 INTRODUCTION

The complex interplay among protein, carbohydrates, and fats significantly influences appetite, energy intake, and overall health. Notably, Western diets, characterised by high levels of industrially processed foods containing “empty calories,” refined sugar, salt, and saturated fat, along with low protein and fibre, have dominated food systems, contributing to overnutrition, obesity, comorbidities, and increased all‐cause mortality (Ludwig & Ebbeling, 2018; Schnabel et al., 2019).

In contrast, diets mainly consisting of minimally processed, especially whole‐food diets, are linked to better health outcomes. These positive effects are often associated with increased intake of plant‐derived foods (PDF) (Song et al., 2016), encompassing essential components like fibre, micronutrients, phytochemicals, complex carbohydrates, and plant proteins, which are typically lacking in industrially processed diets.

Preclinical, clinical, and observational data have demonstrated the importance of both the quality and ratios of macronutrients in the diet on appetite and metabolic outcomes. Comparisons between plant and animal source proteins, as well as the balance of fats versus carbohydrates in counterbalancing protein intake, have been prominent topics in nutritional literature. Diets low‐to‐moderate in protein and higher in complex carbohydrates have shown significant metabolic and lifespan benefits (Solon‐Biet et al., 2014; Wali et al., 2021).

The current study aims to assess the main and interactive effects of protein‐source (plant vs. animal) and fat to carbohydrate ratio, using a 2 × 2 factorial dietary design. We evaluated the impact of these diets on protein appetite and energy intake as well as various health parameters in older individuals. We hypothesised that consumption of a high carbohydrate, low fat plant‐based diet (PBD) will result in favourable health outcomes. Additionally, we report on the overall health benefits associated with transitioning from a standard Australian diet to a whole food diet to evaluate the impact of overall diet quality improvement.

## 2 METHODS

### 2.1 Participants eligibility

**FIGURE 1:**
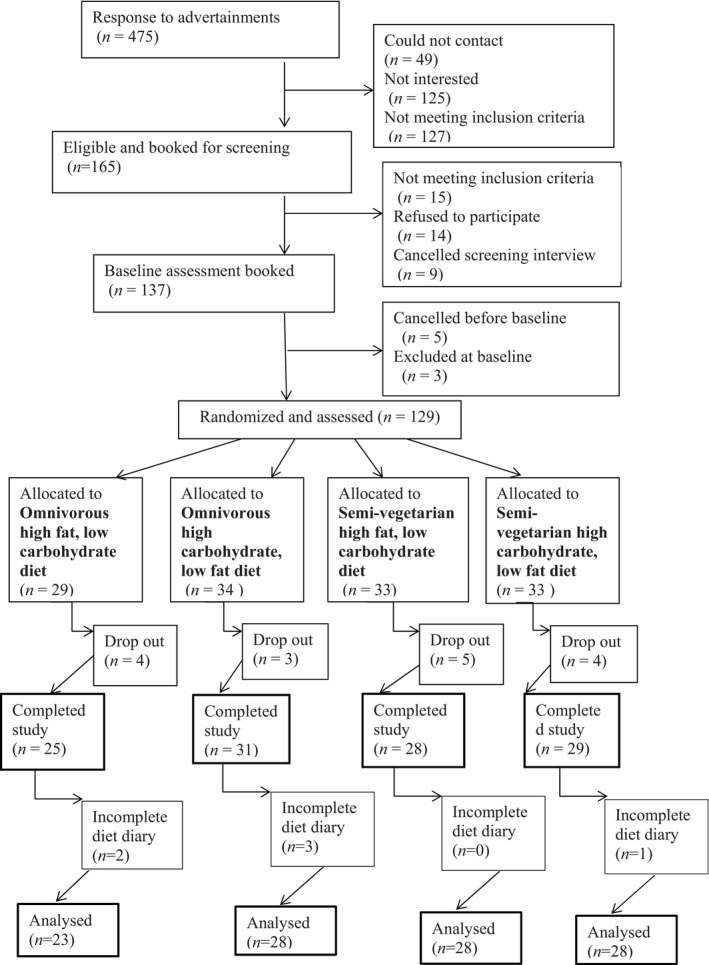
Consort flow diagram for recruitment, randomisation, data collection and analysis.

The study design, recruitment process, eligibility criteria and clinical assessments have been described in detail in the study protocol (Ribeiro et al., 2019). In summary, 113 healthy individuals aged 65–75 years, who were non‐smokers, non‐vegetarians, and had no history of food allergies/intolerances or any health condition known to affect dietary intakes participated in the study (Figure 1). Socio‐demographic data such as marital status, country of birth, level of education and income were collected through questionnaires prior to baseline assessment.

### 2.2 Study design

The study was a 4‐week randomised controlled trial that followed a 2 × 2 factorial design where both the source of dietary protein (pro‐vegetarian vs. omnivorous, at fixed 14% of energy) and the ratio of carbohydrate to fat in the remaining energy complement were evaluated to examine their effects on health. Participants were provided with ad libitum access to one of the following four diets: (a) omnivorous, high fat (OHF); (b) omnivorous, high carbohydrate (OHC); (c) semi‐vegetarian, high fat (VHF); and (d) semi‐vegetarian, high carbohydrate diet (VHC). Detailed dietary compositions of each intervention group are presented in Appendix S1. Diets were matched for energy, protein concentration, and followed the same menu (Appendix S2) with items tailored to each dietary treatment. All foods were prepared by and distributed via a meal delivery company. Participants were asked to consume exclusively the provided food during the 4‐week intervention. Recruitment and data collection processes occurred from April 2017 to June 2018. The primary outcomes were energy intake and protein appetite, measured via food intake and plasma FGF‐21 levels, respectively. Body composition, gut microbiome, muscle strength, physical function, metabolomics, and cardiometabolic measurements were secondary outcomes.

### 2.3 Sample size

Energy intake was used to determine sample size. Using an unpaired, two‐tailed design, a standard deviation of 2.4 MJ, with 80% power and alpha of 0.05 a difference of an average of 1 MJ between any of the four groups may be detected with a sample size of 50 individuals per group. Therefore, considering an estimate drop‐out rate of approximately 10%, a total of 220 participants would be required. A total of 137 participants were recruited, 129 were randomised and 113 completed the study (Ribeiro et al., 2019). At the midpoint of recruitment, a sample size reassessment revealed that the initially planned and approved sample size would not attain statistical significance of the effects observed among dietary treatments. Consequently, the decision was made to terminate recruitment to prevent waste of resources. Participants that had already enrolled at that stage completed the study according to the original plan.

### 2.4 Randomisation

Study coordinators utilised the R package “blockrand” (Snow, 2013) to generate random blocks of eight participants, separately for males and females. The allocation process remained concealed from the participants, ensuring blinding, whilst the study coordinators who enrolled and assigned participants to intervention and conducted assessments and measurements, were unblinded. Data analysis was also unblinded. The sequence of interventions was kept concealed in a sealed opaque numbered envelope until the assignments were made at baseline assessment.

### 2.5 Primary outcome variables

#### 2.5.1 Dietary intake

Participants' dietary intake was determined through collection of weighed food records (WFR). For habitual intake, participants were asked to complete a 7‐day WFR prior to clinical baseline assessment; for intake during the 4‐week study intervention, participants were asked to weigh and record their food intake daily in a study food diary, which contained a list of all meals and snacks (i.e., specific diet menu) supplied by the study (Ribeiro et al., 2019). Individual dietary intakes, both habitual and during the study, were converted into nutrient intakes using FoodWorks 8 Professional for Windows (Xyris Software [Australia] Pty Ltd) with the Australian Food, Supplement and Nutrient Database 2013 (AUSNUT 2013) (Zealand, 2013). Total intake was calculated as the sum of protein, available carbohydrate (sugar and starch), fat and fibre intake in grams. Energy density was calculated as sum of energy derived from protein, available carbohydrate (sugar and starch), fat and fibre intake in kJ divided by total intake in grams.

#### 2.5.2 FGF‐21 concentration

Fasting plasma FGF‐21 concentration (an indicator of protein hunger (Solon‐Biet et al., 2023)) was measured by ELISA (FGF‐21 Quantikine ELISA kit, R&D Systems, USA) according to the manufacturer's instructions and used as a marker for protein appetite as its liver production and plasma concentration are sensitive to protein intake (Hill et al., 2020).

### 2.6 Secondary outcome variables

#### 2.6.1 Body composition, muscle strength, and physical function

Body composition was assessed using air‐displacement plethysmography (BodPod) (COSMED USA, Concord, CA, USA) using a previously described protocol (Ribeiro et al., 2019). Weight and height were used to calculate body mass index (BMI). Waist circumference was measured to the nearest 0.5 cm, at the middle of the lowest rib and the iliac crest, with subjects in standing position and after exhaling. Hip circumference was measured at the point where the buttocks extended the maximum, when viewed from the side. Two consecutive recordings were made for each site to the nearest 1 cm using a metal tape on a horizontal plane without compression of skin. The mean of two sets of values was used. Muscle strength was assessed through handgrip strength using a Jamar dynamometer (Promedics, Blackburn, UK) and chair stand test (time to successfully complete five chair stands using a chair without armrests and a seat height of 40 cm) using the previously described protocol (Ribeiro et al., 2019). Physical function was assessed through walking speed which was measured on a six‐metre course at self‐selected usual pace.

#### 2.6.2 Cardiometabolic markers

Blood pressure levels were obtained using mercury sphygmomanometers on the right arm of seated participants. Systolic and diastolic blood pressures were taken twice to the nearest 2 mmHg and the average recorded. Plasma insulin concentration was measured by ELISA (Human/Canine/Porcine Insulin Quantikine ELISA kit, R&D Systems, USA) according to the manufacturer's instructions. Plasma glucose was assayed in duplicate using an automatic spectrophotometric centrifugal analyser (Roche Hitachi 912, Boehringer Mannheim GmbH, Mannheim, Germany) using the hexokinase/glucose‐6‐phosphate dehydrogenase enzymatic assay. HOMA‐IR (Matthews et al., 1985) was calculated from glucose and insulin data. All plasma and serum measures were assessed in overnight fasting samples.

#### 2.6.3 Targeted metabolomics

Fasting plasma samples were processed using a method described previously (Koay et al., 2021). Briefly, plasma sample was deproteinised using acetonitrile and methanol (75:25; v/v/v) with formic acid (0.2%). A hydrophilic interaction liquid chromatography–tandem mass spectrometry (LC–MS/MS) method was used to the simultaneous detection of 73 metabolites in plasma as previously described (Koay et al., 2021). The LC was connected to an AB Sciex Triple Quad 5500mass spectrometer run in positive ionisation mode. Metabolites peak integration was done on software Multi‐Quant 3.0 for MRM Q1/Q3peak integration. Metabolite peaks with low quality peak morphology and integration were excluded from the analysis. The signal intensity of the ions were log_2_ transformed and the data was normalised by fitting a local regression curve to correct signal drifting as previously described (Kim et al., 2021).

#### 2.6.4 Gut microbiome and faecal short‐chain fatty acid (SCFA) analyses

Stool sample collection and initial processing have been described elsewhere (Ribeiro et al., 2019). DNA was extracted using the FastDNA™ SPIN Kit for Feces (MP Biomedicals #116570200) following the manufacturer's instructions. Amplifiability and concentration of DNA were verified by PCR and Qubit assay kit (Invitrogen). Illumina sequencing of the V4 region (515f‐806r) of the 16S rRNA gene was performed commercially at the Ramaciotti Centre for Genomics (The University of New South Wales). Paired‐end reads (2 × 250bp) were processed with the dada2 package (1.12.1) using R software (3.6.1). Briefly, forward and reverse reads were trimmed (F240; R200) and quality filtered (using default parameters) to remove low sequence bases and error model determined (108 bases). Sequences were then dereplicated and exact amplicon sequence variants (ASVs) inferred before merging of paired‐end reads and removal of chimeric sequence. Taxonomy was assigned using the Ribosomal Database Project (RDP) training set (rdp_train_set_16) with specie level taxonomy (dp_species_assignment_16) assignment (10.5281/zenodo.801828). Exploration of microbiome were performed using the phyloseq package (1.28.0). ASV with mean relative abundance lower than 0.01% were removed from analysis. Alpha diversity of the samples was measured by observed taxa (number of unique ASV), Shannon diversity index, Inverse Simpson index, Evenness and Fisher's alpha index. The observed species index measures the number of different taxa per sample which is defined as “richness”. For beta diversity analysis, a compositional analysis approach was utilised (Gloor et al., 2017). Zero replacement of ASV was first achieved by scaling ASVs with a pseudocount of one before data were centre log‐ratio (CLR) transformed. Difference in overall microbiome composition between treatment groups was determined by PERMANOVA analysis of Aitchison distance. Differentially abundant taxa were determined by ALDEx2 (1.20.0). Plasma acetate levels were measured by nuclear magnetic resonance. Briefly, plasma samples were filtered through a 3 kDa membrane. Polar metabolites were then extracted from the aqueous phase following the addition of deuterium methanol/deuterium chloroform. Samples were run on a Bruker 600 MHz machine with 4,4‐dimethyl‐4‐silapentane‐1‐sulfonic acid as an internal standard and data analysed using the Chenomx Profiler software.

### 2.7 Statistical analysis

Data were analysed in R (v.3.6.1), with significance considered at *p* < 0.05. The analysis involved two main methods: an uncontrolled observation comparing baseline values on a participants' regular diets with the follow up value after the intervention diets using paired sample *t*‐tests (“t.test” function in stats R); and a detailed examination of RCT findings involving all four groups, maintaining treatment assignment integrity, and excluding dropouts.

Diet group analysis utilised a 2 × 2 factorial ANOVA (“aov” function in stats R), fitting main effects of dietary protein source [semi‐vegetarian vs. omnivorous], dietary carbohydrate/fat content [high fat, low carbohydrate vs. high carbohydrate, low fat], and their interaction. A model containing follow‐up measurements as outcomes with adjustments for baseline results was fitted (Model 1) and another version further adjusted for age, BMI, sex, and physical activity level (Model 2) were implemented. The normality of residuals was evaluated visually using histograms; all residuals appeared normally distributed.

Diet‐specific self‐reported appetite and palatability data were analysed using the R package, mgcv using the “gam” function (Wood, 2011). Generalised additive mixed models (GAMMs) were used to investigate the effects of dietary interventions on appetite scores. GAMMs were selected, and using this package, for three reasons. First, we were able to use a beta‐family model (logit‐link), which is designed to analyse response data that fall on a bounded scale between 0 and 1 as our appetite scores do. Second, like more widely known generalised linear mixed model (GLMMs), GAMMs can account for repeated measures (i.e., non‐independence) in the data using random effects; here the same participant completed the questionaries several times per day, on four separate days. All our models included the participant‐date as a random effect. Third, again like GLMMs, GAMMs can include fixed effects alongside random effects. These fixed effects can be conventional parametric terms, which allowed us to test the 2 × 2 effects of plant vs animal protein source and carbohydrate/fat content, and the effects of intervention week (1–4). However, GAMMs can also include non‐linear smooth terms, which allowed to evaluate how appetite scores fluctuate based on the time of the day. To test for interactions between the smooth term for time of day and dietary treatment we built a model that included diet‐specific smooth terms for time of day, and a model that contained a common effect of time of day across all diets. These two models were then compared based on AIC; where the model with diet‐specific smooth terms was favoured we infer the presence of an interaction between time of day and dietary treatment in determining appetite score.

For BCAA levels and metabolites, which are effectively unitless measures, we calculated relative (fold‐change) values as final/baseline. Here the significance of the overall change from baseline was analysed in a one sample t‐test (null hypothesis = 1). The differences in the fold change among the different diets were assessed by fitting a model containing follow‐up measurements as outcomes with adjustments for baseline results, and then another version further adjusted for age, BMI, sex, and physical activity level. For metabolomics, all *p* values were adjusted for the false discovery rate (“p.adjust” function in *stats* R) (Benjamini & Hochberg, 1995).

Dropouts were treated by exclusion (i.e., as opposed to intention to treat, ITT). An ITT analysis was deemed less appropriate in the current case as we sought to directly detect the physiological/metabolic effects of the diet, rather than its efficacy as a population‐wide or clinical intervention. Compliance was assessed by calculating the number of individuals whose mean total energy intake during intervention was >105%; distribution of compliance was then assessed using chi‐squared test. Energy intake served as a metric for compliance as the study supplied all foods (and energy) available to participants. We conducted cross‐checks by comparing statistical compliance with reported cases of extra food consumption obtained during weekly follow‐up calls. An elevated energy intake (>105% of the provided amount) was used to infer that participants had eaten food from external sources beyond the study diets.

We visualise differences from baseline as a function of diet group using Cumming estimation plots (Cumming, 2012). In places we visualise correlation among variables at the individual level via scatter plots and correlation matrices, the latter generated in R using the “corrplot” package (Wei & Simko, 2017).

## 3 RESULTS

### 3.1 Participants

Summarily, from the initial 137 eligible participants, 128 were randomly selected and 113 completed the study (Figure 1). The primary reason for withdrawal was inability to comply with the study diets. No differences were observed in the measured characteristics between completers and non‐completers (Appendix S3). Baseline characteristics and dietary intake were well‐matched across all dietary groups (Table 1). After excluding six participants with incomplete baseline diet diaries, the final sample size for dietary intake analysis was 107. Among these, 23 participants were allocated to the OHF diet, 28 to the OHC diet, and 28 to each of the VHF and VHC diets.

**TABLE 1:** Participants' baseline characteristics and dietary intake by randomly assigned diet treatment group.

		Omnivorous		Pro‐vegetarian	
	Overall, *n* = 113 [*n* = 107]	High fat, low carbohydrate, *n* = 25 [*n* = 23]	Low fat, high carbohydrate, *n* = 31[*n* = 28]	High fat, low carbohydrate, *n* = 28 [*n* = 28]	Low fat, high carbohydrate, *n* = 29 [*n* = 28]
Baseline characteristics					
Age, years	69.4 (2.85)	69.4 (2.9)	69.3 (3.1)	69.3 (2.7)	69.5 (2.8)
BMI, kg/m^2^	27.5 (3.2)	28.2 (3.1)	27.9 (2.9)	26.9 (3.7)	27.0 (3.2)
PASE, points	87.7 (36.1)	80.0 (35.2)	101.1 (39.8)	96.4 (39.5)	73.4 (27.1)
Females, *n* (%)	75 (66)	16 (64)	20 (65)	19 (68)	20 (69)
Married/de facto, *n* (%)	60 (46.9)	15 (52)	13 (38)	20 (61)	12 (38)
High education, *n* (%)	53 (47)	15 (60)	16 (52)	13 (46)	9 (31)
State age pension only, *n* (%)	20 (18)	5 (20)	7 (23)	5 (18)	3 (10)
Ex‐smoker, *n* (%)	68 (60)	14 (56)	15 (48)	18 (64)	21 (72)
Australian‐born, *n* (%)	59 (53)	16 (64)	16 (53)	11 (41)	16 (55)
No. of comorbidities	1.8 (1.5)	1.8 (2.0)	1.5 (1.0)	2.2 (1.4)	1.8 (1.7)
Excellent/good/fair SRH, *n* (%)	107 (98)	25 (100)	26 (93)	27 (100)	29 (100)
Baseline dietary intake					
Energy, kJ	7842 (2021.5)	7476 (2464.3)	7448 (1747.7)	8423 (1884.9)	7954 (1962.4)
Protein, g	85 (22.2)	83 (30.2)	83.1 (17.6)	87 (19.6)	87 (22.1)
Protein, %E	19 (3.5)	19 (3.7)	19 (3.5)	18 (3.4)	19 (3.3)
CHO, g	184 (56.1)	166 (54)	181 (55.3)	192 (54.0)	193 (59.8)
CHO, %E	39 (6.3)	37 (6.8)	40 (5.6)	38 (6.3)	40 (6.5)
Fat, g	76 (24.8)	75 (32.4)	70 (17.4)	83 (25.0)	77 (23.6)
Fat, %E	36 (5.1)	37 (6.2)	35 (4.3)	36 (5.2)	36 (4.9)
Fibre, g	25.4 (9.2)	23.4 (8.4)	25.1 (9.2)	27.1 (10.6)	25.6 (8.3)
Alcohol, g	8.7 (17.2)	10.1 (16.9)	5.6 (9.0)	13.8 (25.9)	5.5 (11.4)

*Note*: Data are presented as means and standard deviation. Square brackets show sample size for dietary intake data. State age pension only category refers exclusively to participants whose income derived is derived solely from the state age pension. High education includes any education obtained after high school, low education includes those who completed high school or below.

Abbreviations: %E, percentage energy; PASE, physical activity for the elderly score; SRH, self‐rated health.

### 3.2 Dietary intake

**FIGURE 2:**
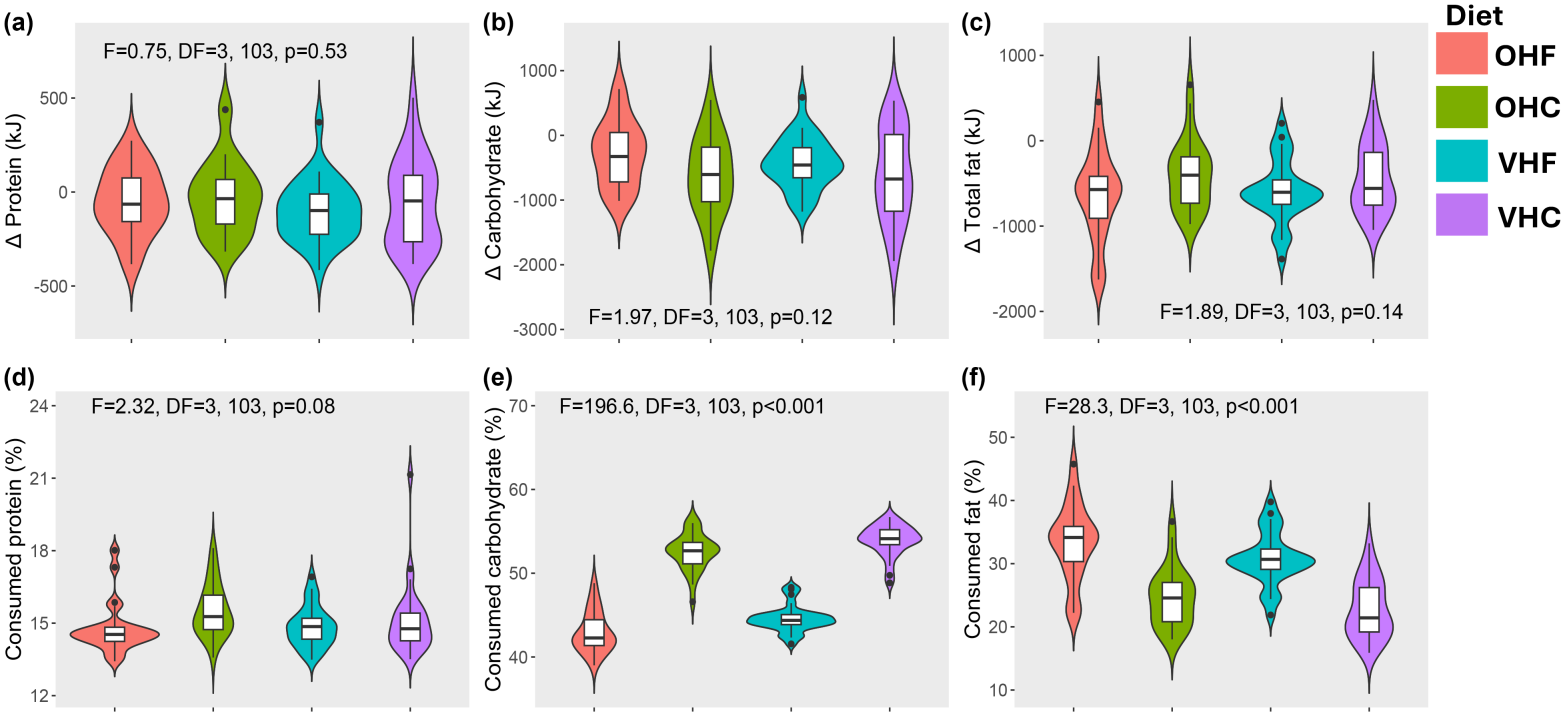
Dietary compliance and macronutrient intake; Plots showing the difference between consumed and available macronutrients (a–c). These plots show that macronutrients consumption was not different from what was offered in the diet. Plots (d–f) show that consumed protein (as percentage of energy) was similar across all dietary groups, whereas carbohydrate and fat differed by group which is in line with the dietary intervention. Statistics derived from ANOVA fitting diet as a four‐level category.

**FIGURE 3:**
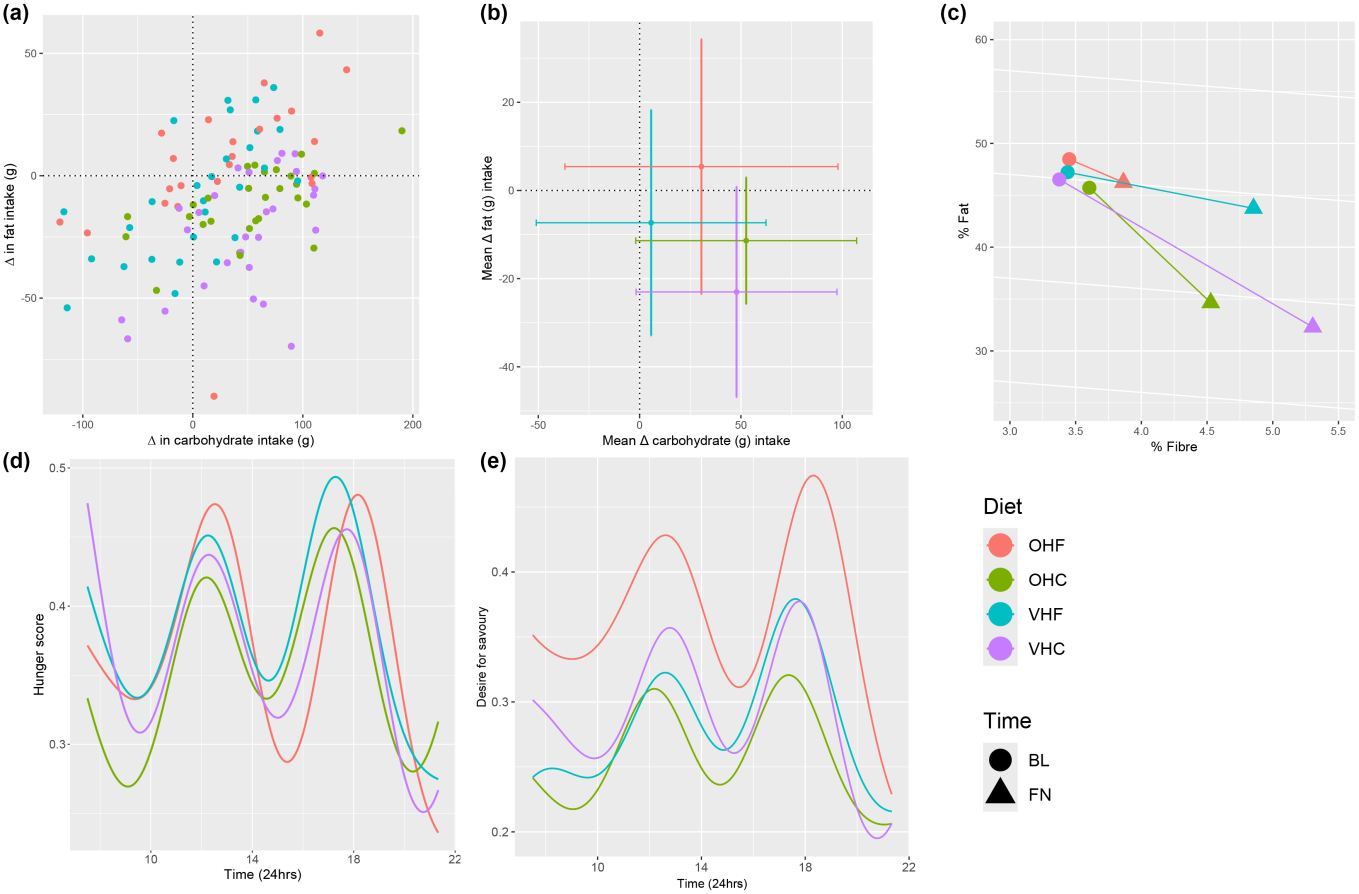
Changes in dietary intake resulted from dietary intervention and effect of intervention on self‐reported appetite measures; changes in carbohydrate (x‐axis) and fat (y‐axis) intake (g) according to diet (a); bivariate mean change in carbohydrate (x‐axis; ± SD as horizontal lines) and fat (y‐axis; ± SD as vertical lines) intake (g) according to diet (b); proportional change in fat, fibre and carbohydrate (%E); %E carbohydrate implicit; %E protein not included as it was the same for all groups (14%E) (c). Absolute carbohydrate intake was reduced in all diets, absolute fat intake was increased in all diets but OHF. Those randomised to the OHF diet had a significantly higher desire for savoury food scores (d). Hunger and desire for savoury foods (e) followed a very similar pattern throughout the day. BL, baseline assessment; FN, final assessment; OHC, omnivorous high carbohydrate; OHF, omnivorous high fat; VHC, semi‐vegetarian high carbohydrate; VHF, semi‐vegetarian high fat.

Ten percent of participants exceeded the provided energy by at least 5%, however compliance did not differ between groups (*χ* ^2^ = 1.73, *p* = 0.63). Macronutrient intake from the study diets was similar among participants (Figure 2a–c). Macronutrient proportions (%E) aligned with the dietary interventions; protein intake was constant across groups (Figure 2d), carbohydrate and fat elevated in their respective high‐exposure groups (Figure 2e,f). However, changes in absolute carbohydrate and fat intakes relative to baseline were small and did not align with expectations for all diets, including high‐carbohydrate diets (Figure 3a,b).

All groups exhibited increased consumption of unprocessed foods resulting in elevated fibre intake. Conversely, there was a decrease in dairy products, animal‐based protein, and highly processed foods, even within omnivorous diets (Appendix S1). Changes from baseline for all treatment groups combined are listed in Table 2. Energy density (*p* < 0.001) and alcohol intake (*p* < 0.001) exhibited notable decline, whereas total energy intake exhibited only a marginal change (113.6 kJ difference from baseline, *p* = 0.06). Protein intake decreased by 20%, likely contributing to the observed decline in urinary urea concentration (a decrease of 62.9mml/L from baseline, *p* < 0.001). In contrast, dietary fibre intake increased by 44%. The VHC group exhibited the largest change in fibre intake, whilst the OHF group showed the smallest changes (Figure 3c).

**TABLE 2:** Baseline versus final measures across all diets pooled, with further assessment of the 2 × 2 effects of protein source and F:C.

	Mean values		Difference	*p* value (2 × 2 analysis)			
	Bl	Fn	Mean (%)	Overall	Protein source	F:C	Interaction
Dietary intake and appetite							
Total intake, g^1^ (*n* = 107)	371.1	389.6	18.4 (5)	**0.04**	0.67 [0.75]	**0.04** [0.18]	0.74 [0.46]
Total energy, kJ (*n* = 107)	7782	7669	−113.6 (−2)	0.6	0.65 [0.73]	0.51 [0.28]	0.61 [0.35]
Macronutrients energy^2^, kJ (*n* = 107)	7390	7321	−69 (−1)	0.7	0.67 [0.75]	**0.04** [0.18]	0.74 [0.46]
Energy density, kJ/g (*n* = 107)	20.5	19. 7	−0.9 (−4)	**<0.001**	**<0.001 [<0.001**]	**<0.001 [<0.001**]	**0.01** [**0.05**]
Energy density exc. alcohol, kJ/g (*n* = 107)	20.5	19.6	−0.9 (−4)	**<0.001**	**<0.001 [<0.001**]	**<0.001 [<0.001**]	0.17 [0.32]
Protein, g (*n* = 107)	85.1	68.0	−17.2 (−20)	**<0.001**	0.45 [0.64]	0.63 [0.96]	0.82 [0.93]
Carbohydrate, g (*n* = 107)	185.1	219.4	34.3 (19)	**<0.001**	0.48 [0.67]	<0.001 [<0.001]	0.64 [0.29]
Total fat, g (*n* = 107)	75.6	65.8	−9.8 (−13)	**<0.001**	**0.02** [0.10]	**<0.001 [<0.001]**	0.72 [0.54]
Fibre, g (*n* = 107)	25.3	36.4	11 (44)	**<0.001**	**<0.001 [<0.001**]	0.003 [0.04]	0.99 [0.84]
Alcohol, kJ (*n* = 107)	217.3	15.1	−202.2 (−93)	**<0.001**	0.19 [0.39]	0.12 [0.40]	**0.01** [**0.02**]
P:C, g (*n* = 107)	0.50	0.31	−0.2 (−36)	**<0.001**	0.01 [0.17]	<0.001 [<0.001]	0.65 [0.21]
FGF‐21, pmol/L (*n* = 103)	215.9	270.9	55 (26)	**<0.001**	0.38 [0.69]	0.86 [0.65]	0.97 [0.93]
BCAA	1	0.99	−0.01 (−0.5)	**0.01**	0.96 [0.98]	0.98 [0.73]	0.82 [0.73]
Body composition							
Body weight, kg (*n* = 107)	74.2	72.4	−1.8 (−3)	**<0.001**	**0.05** [0.10]	0.86 [0.73]	0.60 [0.91]
Fat mass, kg (*n* = 107)	28.5	27.0	−1.5 (−5)	**<0.001**	0.60 [0.52]	0.85 [0.47]	0.88 [0.94]
Fat free mass, kg (*n* = 107)	45.7	45.4	−0.3 (−0.7)	**0.01**	0.35 [0.33]	0.60 [0.48]	0.70 [0.61]
Fat mass, % (*n* = 107)	38.3	37.1	−1.1 (−3)	**<0.001**	0.77 [0.96]	0.81 [0.47]	0.84 [0.83]
Fat free mass, % (*n* = 107)	61.7	62.9	1.1 (2)	**<0.001**	0.77 [0.97]	0.76 [0.43]	0.87 [0.79]
Waist, cm (*n* = 78)	91.2	89.2	−2 (−2)	0.17	0.88 [0.79]	0.76 [0.57]	0.78 [0.79]
Muscle strength							
Grip strength, kg (*n* = 107)	27.9	28.4	0.5 (2)	0.11	0.39 [0.23]	0.70 [0.47]	0.31 [0.17]
Walk speed, m/s (*n* = 107)	5.3	5.29	−0.01 (−0.2)	0.9	0.38 [0.63]	0.76 [0.77]	0.71 [0.95]
Chair stand, s (*n* = 106)	11.7	10.9	−0.8 (−7)	**<0.001**	0.43 [0.40]	0.75 [0.39]	0.96 [0.57]
Cardiometabolic and urinary measures							
SBP, mm Hg (*n* = 107)	134.3	127.0	−7.4 (−5.5)	**<0.001**	0.07 [0.020]	0.17 [0.30]	0.09 [0.14]
DBP, mm Hg (*n* = 107)	78.1	75.6	−2.4 (−3)	**<0.001**	**0.03** [**0.04**]	0.71 [0.61]	0.80 [0.62]
Cholesterol, mmol/L (*n* = 104)	5.5	4.9	−0.7 (−12)	**<0.001**	**0.03** [0.10]	0.63 [0.71]	0.42 [0.46]
LDLc, mmol/L (*n* = 104)	3.6	3.2	−0.4 (−11)	**<0.001**	0.99 [0.77]	0.07 [0.17]	0.52 [0.48]
HDLc, mmol/L (*n* = 104)	1.5	1.36	−0.1 (−7.5)	**<0.001**	0.33 [0.61]	0.82 [0.58]	0.78 [0.89]
Triglycerides, mmol/L (*n* = 104)	1.1	1.2	0.06 (6)	0.36	0.56 [0.18]	0.82 [0.52]	0.69 [0.40]
Glucose, mmol (*n* = 103)	5.5	5.4	−0.1 (−1.6)	0.07	1.00 [0.37]	0.03 [0.18]	0.47 [0.95]
Insulin, pmol (*n* = 103)	60.4	53.8	−6. 6 (−11)	**<0.001**	0.26 [0.35]	0.62 [0.62]	0.13 [0.19]
HOMA‐IR (*n* = 103)	1.15	1.0	−0.1 (−11)	**<0.001**	0.25 [0.34]	0.56 [0.57]	0.14 [0.21]
Urea concentration, mmol/L (*n* = 106)	249.1	186.3	−62.9 (−25)	**<0.001**	0.11 [0.24]	0.78 [0.89]	0.61 [0.86]
Gut microbiome and SCFA							
Diversity observed (*n* = 102)	138.7	143.8	5.1 (4)	0.27	0.31 [0.34]	0.95 [0.59]	**0.04** [**0.03**]
Diversity Shannon (*n* = 102)	3.9	3.9	0.07 (2)	0.18	0.26 [0.39]	0.75 [0.94]	**0.01** [**0.01**]
Diversity InvSimpson (*n* = 102)	28.4	30.5	2.1 (7)	0.24	0.45 [0.62]	0.86 [0.82]	**0.004** [**0.004**]
Diversity Fisher (*n* = 102)	17.9	18.6	0.7 (4)	0.33	0.28 [0.32]	0.99 [0.57]	**0.04** [**0.02**]
Diversity evenness (*n* = 102)	0.4	0.4	0.01 (2)	0.23	0.18 [0.32]	0.76 [0.94]	**0.01** [**0.01**]
Firmicutes (*n* = 102)	75.2	76.4	1.3 (2)	0.38	0.38 [0.36]	0.95 [0.70]	0.10 [0.20]
Verrucomicrobia (*n* = 102)	3.1	3.3	0.3 (9)	0.70	0.37 [0.41]	0.85 [0.63]	0.62 [0.43]
Actinobacteria (*n* = 102)	7.4	6.6	−0.8 (−11)	0.40	0.35 [0.59]	**0.04** [0.07]	0.76 [0.82]
Bacteroidetes (*n* = 102)	10.7	10.7	0.07 (0.7)	0.94	0.70 [0.29]	0.21 [0.39]	0.42 [0.99]
Proteobacteria (*n* = 102)	2.3	1.4	−0.9 (−38)	0.12	0.54 [0.41]	0.18 [0.27]	0.06 [**0.03**]
Euryarchaeota (*n* = 102)	1.3	1.4	0.1 (6)	0.80	0.15 [0.21]	0.11 [0.13]	0.93 [0.85]
Synergistetes (*n* = 102)	0.01	0.02	0 (43)	0.56	0.12 [0.11]	0.73 [0.49]	0.80 [0.59]
Acetate (*n* = 94)	71.9	79.7	7.8 (11)	**0.02**	1.00 [0.81]	0.14 [0.19]	0.13 [0.06]

*Note*: ^1^Total intake was calculated as the sum of protein (g), carbohydrate (g), fat (g) and fibre (g) intake; ^2^macronutrients energy (kJ) is the sum of energy derived from protein, carbohydrate and fat; P:C was calculated as protein (g) divided by carbohydrate (g); energy density was calculated as sum of macronutrient energy derived from protein, carbohydrate, fat and fibre intake in kJ divided by total intake in grams. *p* values for Model 1 (follow‐up adjusted for baseline assessment) are presented, whilst values in square brackets represent *p* values derived from Model 2 (further adjusted for age, BMI, sex, and physical activity level). Bold values highlight statitistically significant results.

Abbreviations: BCAA, branched‐chain amino acids; BL, baseline assessment; DBP, diastolic blood pressure; F:C, fat‐to‐carbohydrate ratio; FGF‐21, fibroblast growth factor 21; FN, final assessment after 4‐week intervention; HDLc, high‐density lipoprotein; HOMA‐IR, Homeostatic Model Assessment for Insulin Resistance; LDLc, low‐density lipoprotein cholesterol; P:C, protein to carbohydrate ratio; SBP, systolic blood pressure.

### 3.3 Appetite and palatability scores

Median appetite scores were similar across all groups, except for “desire for savoury foods” which was highest on the OHF diet, yielding a significant interaction between protein source and C:F (*p* < 0.001, Figure 3e, Appendix S4). Hunger and desire for savoury foods followed similar and predictable temporal patterns (Figure 3d,e). Median palatability scores for pleasantness, taste, satisfaction, and enjoyment were consistently high across all diets (Appendix S4).

### 3.4 FGF‐21 levels

**FIGURE 4:**
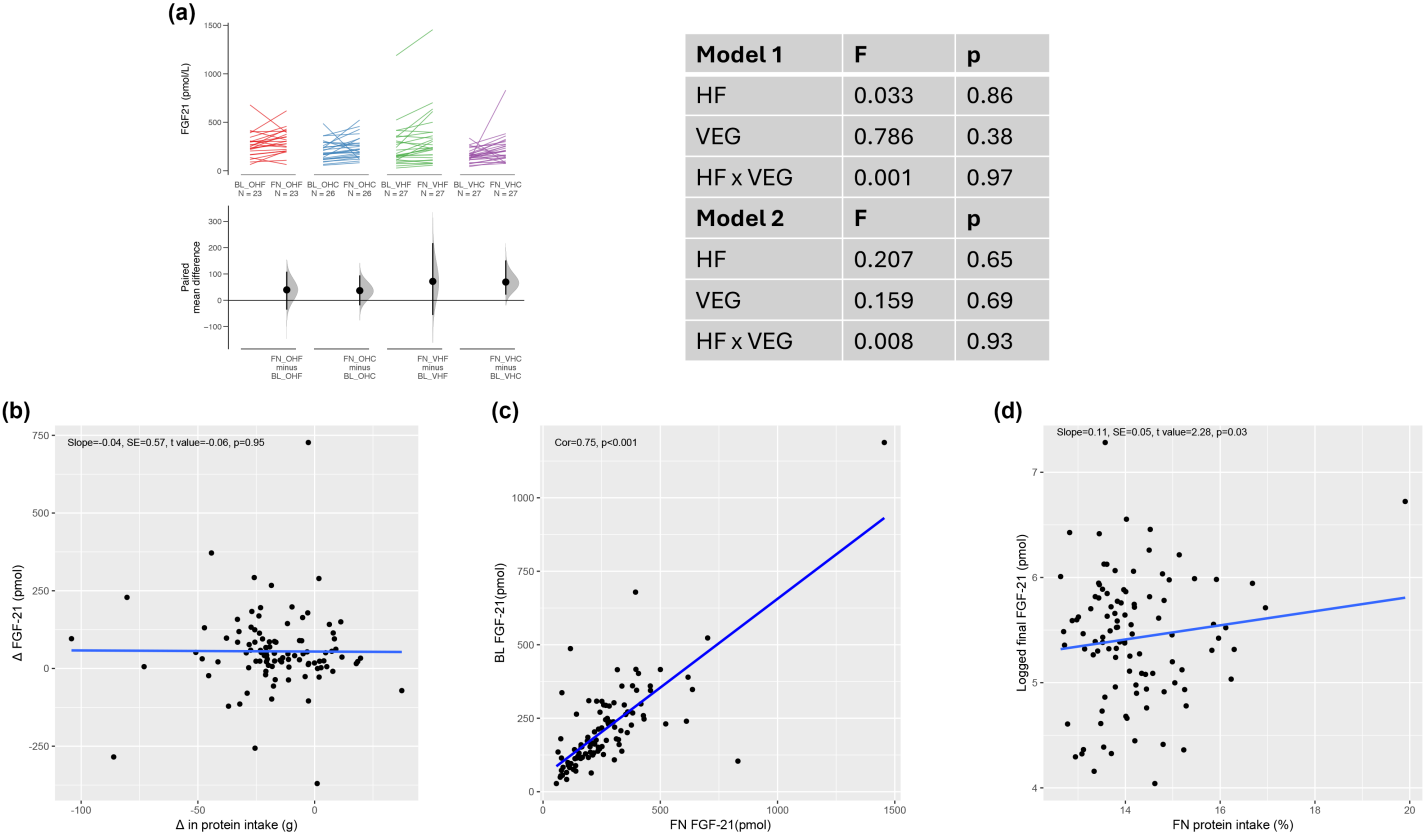
Predictors of change in FGF‐21 levels; Cumming plots showing changes FGF‐21 (a) by diet. Scatter plots showing the relationship between changes in FGF‐21 levels and change in protein intake (b), correlation between baseline and final FGF‐21 (c) and relationship between logged final FGF‐21 and final protein intake (d). Error bars are 95% confidence intervals found by bootstrapping. BL, baseline assessment; FN, final assessment; OHC, omnivorous high carbohydrate; OHF, omnivorous high fat; VHC, semi‐vegetarian high carbohydrate; VHF, semi‐vegetarian high fat.

An overall 25.5% increase in FGF‐21 was observed after intervention (Table 2), commensurate with the decline in protein intake across all treatments. The increase in FGF‐21 was slightly greater among those on pro‐vegetarian diets (Figure 4a), though protein source did not have an effect (*p* = 0.69 fully adjusted model). Individual changes in FGF‐21 were not predicted by changes in protein intake (Figure 4b). However, baseline and final FGF‐21 levels were strongly correlated (Figure 4c), as were final protein (%E) and FGF‐21 levels (Figure 4d). Additionally, individuals with higher FGF‐21 levels during both assessments tended to selectively consume higher protein study foods or supplement their intake with non‐study foods (milk and meat‐based discretionary foods). Analysis of discarded portions of the provided menus revealed that the protein content of consumed foods was higher than that of discards (14.6% vs. 13.3%, *t* = 2.94, df = 106, *p* = 0.004). There were no differences between diet groups in this regard (data not shown).

### 3.5 Body composition and muscle function

**FIGURE 5:**
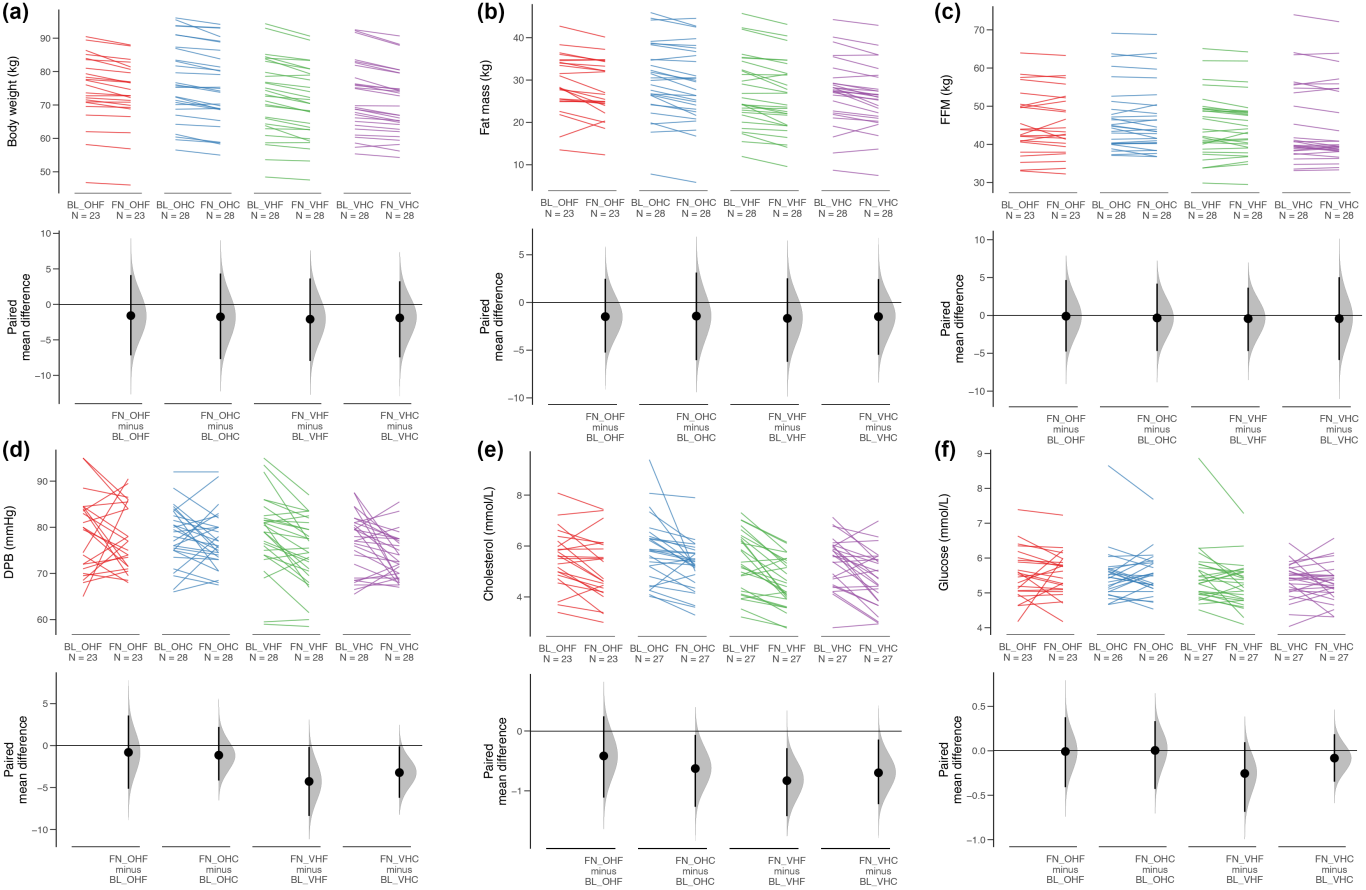
Predictors of change in body composition and selected markers of cardiometabolic health; Cumming plots showing body weight (a), fat mass (b), fat free mass (c), diastolic blood pressure [DP] (d), cholesterol (e) and glucose levels (f) by diet. Error bars are 95% confidence intervals found by bootstrapping. BL, baseline assessment; FN, final assessment; OHC, omnivorous high carbohydrate; OHF, omnivorous high fat; VHC, semi‐vegetarian high carbohydrate; VHF, semi‐vegetarian high fat.

Based on overall uncontrolled observations analysis, there was a 3% reduction in body weight (*p* < 0.001), 5% reduction in fat mass (FM) (*p* < 0.001), and a 0.7% reduction in fat free mass (FFM) (*p* = 0.01, Table 2, Figure 5a–c). Weight loss was recorded in 100 out of 107 (94%) participants with complete data. The degree of weight lost appeared to be higher amongst those on pro‐vegetarian diets, however, protein source effect was no longer present in the fully adjusted model (Figure 5a, Appendix S5). Furthermore, an average weight loss of 1.7 kg was observed in the week preceding baseline assessments whilst WFRs were being completed. Despite the small reduction of FFM, which includes muscle mass, there was no change in grip strength nor walking speed, but a statistically significant 7% reduction in chair stand time post‐intervention, which was not diet‐specific (Table 2, Appendices S5 and S6). Protein source, carbohydrate/fat content, and their interaction did not affect the reduction in waist circumference (Table 2, Appendix S5).

### 3.6 Cardiometabolic parameters

Overall, in the uncontrolled observation analysis comparing baseline to follow‐up, there was a reduction in systolic (SBP) and diastolic blood pressure (DBP), as well as total‐, LDL‐, and HDL‐cholesterol, insulin, and HOMA‐IR at follow‐up assessment (Table 2). The reduction in DBP was greatest for those randomised to pro‐vegetarian diets even after adjustment for sex, age, BMI, and physical activity level (*p* = 0.04, Figure 5d, Table 2 and Appendix S5). There was an effect of protein source on total cholesterol (*p* = 0.03) and glucose levels (*p* = 0.03); however, this effect was no longer present in the fully adjusted model (*p* = 0.18 and *p* = 0.10 respectively, Figure 5e,f, Table 2 and Appendix S5).

No effect of protein source, carbohydrate/fat content, nor their interaction were observed on reductions in SBP, HDL and LDL cholesterol, triglycerides, insulin, and HOMA‐IR measures (Appendices S5 and S7).

### 3.7 Gut microbiome composition and SCFA production

The dietary intervention resulted in changes in the measures of alpha diversity of the gut microbiome (Table 2). The findings suggest that those on the OHC diet experienced an increase in all indices of gut microbiome diversity. All measures of gut microbiome diversity were negatively associated with weight loss (Appendix S8). At the individual level, there was considerable heterogeneity in the response of gut microbiome diversity to the intervention on all diets (Appendix S9). Differential abundance analysis testing by ALDEx2 revealed no significant taxa (at the ASV level) between any of the diets post‐intervention. At the Phylum level, there was change in relative abundance of Actinobacteria, which appeared to be F:C ratio dependent (*p* = 0.04, Table 2 and Appendix S5) but was no longer present in the fully adjusted model (*p* = 0.07, Table 2 and Appendix S5). On the other hand, the relative abundance of Proteobacteria increased amongst those on the OHF, an effect only evident in the fully adjusted model (*p* = 0.03, Table 2, Appendix S9). Change in Fusobacteria was inversely correlated with change in HOMA‐IR, whereas change in Firmicutes and Proteobacteria were positively associated with changes in HOMA‐IR (Appendix S8). There was an increase in plasma acetate levels post‐intervention (79.7 at follow‐up vs 71.9 at baseline, *p* = 0.02, Table 2) which was higher amongst those who consumed the VHC diet (*p* = 0.06, Appendices S4 and S8).

### 3.8 Plasma metabolites

In the uncontrolled observation analysis comparing baseline to follow‐up, circulating BCAA levels decreased by 0.5% after intervention (*p* = 0.01, Table 2). There were, however, no differences in the degree of change in circulating BCAAs between diet groups (Appendix S10). There was a decline in valine, aspartate, glutamine butyrylcarnitine, cysteamine, cytidine, kynurenic acid, 3‐deazaadenosine, 3‐indolepropionic acid, N‐acetyl glutamine, isovalerylcarnitine and an increase in the circulating level of aspartate and glutamine post‐intervention (Appendix S10). Aspartate, adenosine, taurine and triiodothyronine levels were affected by protein source, L‐homoserine by F:C ratio and cytidine levels were affected by both protein source and F:C ratio. However, enrichment analysis did not identify any noteworthy pathways (data not shown).

## 4 DISCUSSION

To our knowledge, this is the first study to investigate the effects of protein source and carbohydrate/fat ratios in older individuals. The data analysis was conducted using two approaches: first, a detailed examination of RCT findings involving all four groups, maintaining treatment assignment integrity but excluding dropouts; and second, an uncontrolled observation comparing participants' usual diet with the intervention diets. Following the 4‐week intervention, participants experienced improvement across all measured health domains independent of diet treatments. This is likely explained by the transition from a typical Australian diet to a less processed diet containing whole foods, such as fruit and vegetables, and limiting intake of highly processed foods and alcohol. Another common factor across diet treatments was a reduction in protein, from a baseline of 19% of total energy to 14% in the experimental treatments. Reduced dietary protein is predicted to improve markers of cardiometabolic health, such as reducing BCAA levels, increasing FGF‐21 concentration, and improving glucose tolerance and insulin sensitivity, particularly in middle‐aged individuals (Fontana et al., 2016). Additionally, it is suggested to expend lifespan (Le Couteur et al., 2016). Many of these changes were seen. A decrease in circulating BCAA was to be expected with a reduction in protein intake (S. M. Solon‐Biet et al., 2022). Likewise, an increase in FGF‐21 levels, a known signal of reduced protein status, is to be predicted.

### 4.1 Appetite, dietary intake and FGF‐21 levels

Whilst total energy intake and most appetite scores did not change during the intervention, significant changes were observed in FGF‐21 levels. Protein restriction increases circulating FGF‐21 levels and increases protein appetite (Laeger et al., 2014). In our study, we found that a 21% reduction in protein consumption led to a 25% increase in FGF‐21 levels. Typically, a decrease in protein percentage (%P) is accompanied by a compensatory increase in total food intake (protein leverage) (Simpson & Raubenheimer, 2005). In the current study we observed a 5% increase in overall food intake, but this was insufficient to attain the same absolute protein intakes as those at baseline. Subjects with high FGF‐21 levels at baseline continued to have a high appetite for protein during intervention and were more likely to consume high‐protein foods outside of the dietary treatment (e.g., milk and meat). Participants preferentially selected high‐protein items within the provided meals. This result is intriguing, especially given that participants were not consuming large quantities of other low‐energy, low‐protein foods. It aligns with the emerging understanding that “protein leverage” is particularly prominent in environments dominated by unhealthy food options (Grech et al., 2022).

### 4.2 Body composition

The observed changes in body composition without a significant change in energy consumption was intriguing. Even before the intervention, participants experienced an average weight loss of 1.7 kg whilst keeping the baseline 7‐day weighed food record (WFR) (Ribeiro et al., 2019). WFRs are considered gold standard for dietary assessment and are believed to be the most accurate method for self‐measuring intake, but can be laborious to complete (Shim et al., 2014). Several studies have shown that recording dietary intake, even in WFRs, may result in unintentional improvement in diet (Burke et al., 2011), and that individuals may also eat less food to minimise the burden of recording (Shim et al., 2014). Both mechanisms may result in individuals eating fewer calories whilst undertaking a WFR. That being the case, any differences between reported baseline and reported final energy intake maybe an underestimate of the true reduction in energy intake, and thus not directly reflect the ultimate weight loss arising from participation in the study.

Weight loss may also be linked to the significant reduction in alcohol intake observed during the intervention. High alcohol consumption (>3 standard drinks) was an exclusion criterion. Nonetheless, alcohol consumption during intervention was discouraged and as a result alcohol intake was reduced by an average of 202.5 kJ/day which could explain, in part, the weight reduction.

Interestingly, weight and fat loss were accompanied by modest reduction of FFM, yet this did not affect muscle strength. Instead, it led to an improvement in chair stand test, a marker of muscle function. FM accounts for overall body fat, including total visceral, subcutaneous fat, as well as intermuscular adipose tissue. Reduction in adipose tissue, rather than gains or mitigated losses in muscle mass, is important in improving function in older age (Santanasto et al., 2011). In fact, it has been suggested that older adults can improve function whilst simultaneously losing both fat and muscle mass, provided that the individual loses a significantly greater proportion of fat mass compared to muscle mass (Santanasto et al., 2011), which was the case in the current study.

### 4.3 Adherence to diets

Transitioning from an omnivorous to a plant‐based diet is thought to be difficult. However, we have shown here that adherence to PBD were no worse than omnivorous diets, and participants' palatability scores were generally high across all dietary interventions. Previous studies have found adherence and acceptability of PBD of all levels (vegan, vegetarian, pesco‐vegetarian, semi‐vegetarian) to be similarly good (Barnard et al., 2009). Nevertheless, shifting towards a PBD can be made easier if gradual and well‐supported (Haverstock & Forgays, 2012). In the current study, the difference in animal/plant content was only subtle (30% more plant protein in the semi‐vegetarian diets), all meals were provided, study menus were thoroughly discussed at screening and weekly phone support was also provided.

### 4.4 Cardiometabolic health

Our study showed that consumption of pro‐vegetarian diets, regardless of carbohydrate‐to‐fat ratio, resulted in the greatest reduction in diastolic blood pressure. Furthermore, overall participation in the study (i.e., not diet specific) resulted in reductions in SBP, total‐, LDL‐ and HDL‐cholesterol, insulin and HOMA‐IR. Other studies have previously shown the beneficial effect of PBD on cardiometabolic health through reduction in plasma lipids (Barnard et al., 2000), blood pressure (Yokoyama et al., 2014), and increase insulin sensitivity (Hung et al., 2006). For instance, partial replacement of meat with plant protein has been shown to improve insulin sensitivity and total and LDL cholesterol levels in postmenopausal women with abdominal obesity (van Nielen et al., 2014). A number of dietary factors such as reduction in energy, saturated fat, cholesterol as well as increase in fibre, polyunsaturated and monounsaturated fatty acids, antioxidants, micronutrients, vegetable protein, and plant sterols can improve glucose control and blood pressure through improving vasodilation, decreasing blood viscosity, improving insulin sensitivity, altering baroreceptors, modifying both renin‐angiotensin, and sympathetic nervous systems (Alexander et al., 2017; Kahleova et al., 2017). In the current study, all diets were relatively high in plant‐based foods, however, levels of vegetable protein and plant sterols were likely to be higher in the pro‐vegetarian diets. Furthermore, the study diets were formulated to minimise added and refined sugars, sodium, and saturated fats—commonly present in ultra‐processed foods. These factors are recognised to influence HOMA‐IR, insulin levels, and diastolic blood pressure.

Our findings are also directly relevant to the debate around the ‘carbohydrate‐insulin model’(CIM) of obesity, which proposes that high dietary carbohydrate increases post‐prandial insulin secretion, causing increased fat accumulation, increased hunger, excess energy intake and ultimately adiposity and metabolic dysfunction (Ludwig & Ebbeling, 2018). Under this model we might expect to see an effect of dietary fat/carbohydrate ratio on insulin (i.e., participants randomised to low‐fat, high‐carbohydrate diets would experience raised fasting insulin). We did not measure postprandial insulin, however, fasting insulin was not affected by protein‐source nor F:C ratio and adiposity, nor did food intake differ with F:C. Our study is the second to experimentally manipulate dietary F:C, albeit with less extreme carbohydrate and fat content, at similar percent protein (Hall et al., 2021), and the first to do so in free‐living individuals.

### 4.5 Gut microbiome and acetate

Food components (e.g., fibre) which are resistant to human digestion are metabolised by the gut microbiome. Since bacteria are specialised in fermenting different substrates, different dietary components may promote or inhibit the growth of specific phylotypes (Tan et al., 2023) and hence gut microbiome composition and diversity. More digestible carbohydrates as well as protein and fat have also been shown to have an effect on the gut microbiome. For example, David et al. (David et al., 2014) showed that a very short‐term consumption of an animal‐based diet was sufficient to increase the abundance of bile‐tolerant microorganisms and decrease the levels of plant polysaccharide metabolising bacteria.

In the present study, those subjects randomised to the OHC diet experienced the biggest change in gut microbiome diversity. However, plasma acetate levels, an abundant SCFA produced by bacterial fermentation of dietary fibre, were increased in all dietary groups. This is in line with the increase in fibre consumption across all diets. SCFA, such as acetate, have been associated with better health outcomes, conferring protective effects in inflammation, cancer, diabetes and cardiovascular diseases.

Plasma levels of 3‐IPA, a tryptophan metabolite produced by gut bacteria, were significantly increased post‐intervention and were affected by protein source. Previous studies have shown that elevated concentrations of IPA in human blood plasma were correlated with a lower risk of type‐2 diabetes (de Mello et al., 2017). IPA treatment has been reported to improve glucose metabolism and attenuate steatohepatitis (Zhao et al., 2019).

Finally, in terms of gut microbiome composition at the phylum level, only very small changes of Actinobacteria level were observed. Like ours, other short‐term studies were not able induce major microbiome composition changes (Tan et al., 2023). Number of faecal samples, time of collection, small study sample size, failure to account for individuals' medication use and lifestyle are potential reasons for the lack of significant effect of dietary intake on participants' gut microbiome (Zmora et al., 2019). Furthermore, the composition of adult humans' gut microbiome is fairly stable (Lozupone et al., 2012), therefore short‐term interventions are unlikely to have a profound impact on microbiota composition. It is more likely that gene expression and functional profiles promptly adapt to dietary changes (Graf et al., 2015), which is responsible for increase in acetate levels in the current study. Finally, it is important to also note that diet is only responsible for 5–20% of the variation in microbiome (Zmora et al., 2019), with factors such as environmental differences, early life exposures, and other host differences influencing microbiome variation (Johnson et al., 2020).

### 4.6 Limitations

Our study has some limitations. First, several inferences arise from observational analysis rather than the RCT component. This study thus provided an opportunity to explore in a semi‐controlled setting the impact of transitioning dietary intake from a Western, processed diet to a minimally processed, whole food diet on the health of older individuals. Second, the number of faecal samples collected may not have been sufficient to capture gut microbiome changes. Recent studies have suggested that repeated sampling of the microbiome through collection of three to five daily sequential samples may be required to account for intra‐individual variation (Johnson et al., 2019). Third, although compliance was high and similar across all diets, it relied on participants self‐reporting; therefore, we cannot discount the possibility of misreporting. Fourth, our findings are restricted to relatively healthy older Australian adults and may not be generalised to other populations. Fifth, although this was a relatively long‐term controlled feeding study compared to other controlled‐feeding studies lasting a few days, our study would be categorised as a short‐term intervention overall. Therefore, the results should be interpreted accordingly. Finally, we intentionally opted for moderate manipulation in protein and F:C to reflect a maximally feasible dietary intervention, acknowledging the possibility that significant effects may not have been observed as a result.

## 5 CONCLUSION

In conclusion, our observational results suggest that transitioning from a “standard Australian diet” to feasible alternatives‐whether moderately higher or lower in F:C and predominantly plant‐or animal‐based‐can improve several health markers relevant to older age. This demonstrates that a healthy diet rich in fruit, vegetables, fibre, moderate amounts of protein, regardless of their sources, while restricting alcohol and highly processed food, can be beneficial for various health domains in older adults. Furthermore, PBD appears to offer additional benefits for cardiometabolic health. Further research is required to establish long‐term effects of such diets.

## AUTHOR CONTRIBUTIONS

Stephen J. Simpson, Alison Gosby, Rosilene V. Ribeiro and David Raubenheimer designed the study. Alison Gosby wrote the protocol and coordinated ethics. Alison Gosby and Rosilene V Ribeiro coordinated contractual, data management, collaborations arrangements, developed study design, recruitment process and determined outcomes. Rosilene V Ribeiro and Alistair M Senior conducted the statistical analyses. Laurence Macia and Jian Tan conducted gut microbiome analysis. Rosilene V Ribeiro drafted the manuscript, and all authors contributed to and approved the final version of the manuscript.

## FUNDING INFORMATION

This research was funded by a generous philanthropic donation from Emeritus Professor George Palmer, and RVR was supported by a Charles Perkins Centre Early Career Fellowship from Ms Jennie Mackenzie. LM was funded by ARC LP160100627. The funders played no role in the design, execution, analysis, and interpretation of data, or writing of the study.

## CONFLICT OF INTEREST STATEMENT

The authors declare no competing interests.

## Supporting information


Appendix S1.

## Data Availability

The data that support the findings of this study are available on request from the corresponding author. The data are not publicly available due to privacy or ethical restrictions. Statistical analysis R codes are available in a GitHub repository (https://github.com/Rosieribeiro/NHL_analysis).
