# Supplementary material for: Rapid benefits in older age from transition to whole food diet regardless of protein source or fat to carbohydrate ratio: Arandomised control trial
Source: Aging Cell. 2024 Jul 16;23(11):e14276. doi: 10.1111/acel.14276 (PMC11561649; doi:10.1111/acel.14276)
Supplement: Supplementary file 1 — Appendix S1. [file ACEL-23-e14276-s001.docx]

**SUPPLEMENTARY MATERIAL**

| **Appendix 1** **-** Dietary intakes at baseline and during dietary intervention (n=107 with dietary data at baseline and final assessment) | | | | | |
| --- | --- | --- | --- | --- | --- |
| **Diet components** | **Baseline** | **OHF** | **OHC** | **VHF** | **VHC** |
| Energy, kJ | 7782.3 | 7842.4 | 7611.9 | 7660.8 | 7590.9 |
| Protein, g (E%) | 85.1 (19) | 68 (14) | 69 (15) | 67 (14) | 67 (14) |
| Carbohydrate, g (E%) | 185.1 (39) | 197.7 (40) | 234.9 (50) | 200.6 (42) | 240.6 (51) |
| Sugar, g | 81.6 | 82.2 | 106.2 | 81.8 | 107.0 |
| Starch, g | 103 | 115.1 | 128.5 | 117.9 | 133.0 |
| Total fat, g (E%) | 75.6 (36) | 79.6 (37) | 58.1 (28) | 74.0 (36) | 54.0 (26) |
| Saturated fat, g | 28 | 18.3 | 15.9 | 15.1 | 12.3 |
| Polyunsaturated fat, g | 12 | 17.9 | 9.1 | 18.7 | 9.8 |
| Monounsaturated fat, g | 29 | 31.3 | 23.2 | 29.1 | 22.8 |
| Dietary fibre, g | 25 | 30.7 | 34.9 | 37.8 | 41.0 |
| **Micro-nutrients** |  |  |  |  |  |
| Thiamine, mg | 1.5 | 1.0 | 1.3 | 1.1 | 1.4 |
| Riboflavin, mg | 1.8 | 1.3 | 1.7 | 1.3 | 1.7 |
| Niacin, mg | 22.9 | 14.6 | 17.5 | 14.7 | 17.3 |
| Niacin eq, mg | 39 | 27.0 | 30.0 | 26.9 | 29.5 |
| Vitamin C, mg | 94.4 | 127.4 | 126.5 | 125.4 | 130.3 |
| Vitamin E, mg | 13 | 16.2 | 12.0 | 15.4 | 11.8 |
| Tocopherol alpha, mg | 11.1 | 12.6 | 10.8 | 11.9 | 10.4 |
| Vitamin B6, mg | 1.4 | 1.1 | 1.1 | 1.2 | 1.1 |
| Vitamin B12, µg | 4.3 | 2.5 | 2.7 | 1.9 | 2.1 |
| Total folate, µg | 505.7 | 372.9 | 453.8 | 525.7 | 564.2 |
| Folic acid, µg | 178.9 | 417.6 | 506.0 | 570.6 | 619.0 |
| Folate food, µg | 326.8 | 66.7 | 77.7 | 66.9 | 81.7 |
| Folate total DFE, µg | 625.5 | 306.3 | 376.0 | 458.9 | 482.6 |
| Total vit A eq, µg | 927.3 | 1060.6 | 1327.7 | 1017.5 | 1345.5 |
| Retinol, µg | 300.5 | 154.2 | 178.1 | 117.9 | 105.7 |
| Beta carotene eq, µg | 3767.1 | 5438.9 | 6899.0 | 5397.2 | 7442.3 |
| Beta carotene, µg | 3101.8 | 4612.1 | 5898.6 | 4527.8 | 6345.1 |
| Sodium, mg | 2078.5 | 1267.7 | 1294.2 | 1273.6 | 1353.2 |
| Potassium, mg | 3084.9 | 2692.9 | 3221.5 | 3095.3 | 3540.2 |
| Magnesium, mg | 344.5 | 339.7 | 347.9 | 389.2 | 385.7 |
| Calcium, mg | 844.3 | 787.8 | 889.7 | 817.5 | 911.9 |
| Phosphorus, mg | 1438.1 | 1058.2 | 1123.6 | 1253.3 | 1286.7 |
| Iron, mg | 10.8 | 9.1 | 10.4 | 11.7 | 12.9 |
| Zinc, mg | 10.1 | 8.0 | 7.7 | 8.0 | 7.8 |
| Selenium, µg | 85.3 | 40.3 | 44.4 | 41.9 | 44.3 |
| Iodine, µg | 178.6 | 125.9 | 125.9 | 130.5 | 119.6 |
| **Food groups** |  |  |  |  |  |
| Grains | 5 | 5.1 | 5.7 | 4.4 | 5.2 |
| Wholegrains (%) | 42 | 58.6 | 53.5 | 60.6 | 54.1 |
| Fruit | 1.4 | 1.4 | 2.2 | 1.5 | 2.4 |
| Vegetables | 3.7 | 5.0 | 5.5 | 6.6 | 6.6 |
| Legume | 0.1 | 0.1 | 0.1 | 1.5 | 1.1 |
| Dairy | 1.7 | 0.9 | 1.2 | 1.3 | 1.4 |
| Milk | 1.1 | 0.8 | 1.0 | 0.8 | 1.0 |
| Milk alternatives | 0 | 0.0 | 0.0 | 0.3 | 0.3 |
| Protein foods | 2.5 | 1.8 | 1.1 | 2.6 | 1.8 |
| Red meats | 0.6 | 0.3 | 0.3 | 0.1 | 0.1 |
| Poultry | 0.4 | 0.1 | 0.2 | 0.0 | 0.0 |
| Eggs | 0.3 | 0.1 | 0.1 | 0.1 | 0.1 |
| Seafood | 0.4 | 0.1 | 0.1 | 0.1 | 0.1 |
| Nuts/seeds | 0.6 | 1.0 | 0.3 | 1.1 | 0.5 |
| Legumes protein | 0.1 | 0.1 | 0.0 | 1.0 | 0.7 |
| Soy products | 0 | 0.0 | 0.0 | 0.2 | 0.2 |
| Oil equivalents (tsp) | 7.7 | 12.2 | 8.3 | 12.3 | 8.9 |
| Added sugar (tsp) | 6.5 | 2.7 | 1.5 | 2.4 | 1.6 |
| Solid fat (tsp) | 8.6 | 3.3 | 3.6 | 2.4 | 2.3 |
| E%, percentage energy; eq, equivalent. Oil equivalents include fats naturally occurring in nuts, seeds, avocado, seafoods, and unhydrogenated vegetable oils. Solid fat equivalents include fats naturally occurring in meat, poultry, eggs, dairy, fully or partially hydrogenated oils, shortening, palm oil and coconut oil. Added sugars include added forms of dextrose, fructose, sucrose, lactose, sugar syrups and fruit syrups. | | | | | |

**Appendix 2 –** Study menu

|  | **Day 1** | **Day 2** | **Day 3** | **Day 4** | **Day 5** | **Day 6** | **Day 7** |
| --- | --- | --- | --- | --- | --- | --- | --- |
| **Breakfast 1** | Muesli & Yoghurt 1 | Muesli & Yoghurt 1 | Muesli & Yoghurt 2 | Muesli & Yoghurt 2 | Muesli & Yoghurt 1 | Muesli & Yoghurt 2 | Muesli & Yoghurt 2 |
| **Breakfast 2** | Vegetable slice | Toast | Corn Fritters | Toast | Corn Fritters | Toast | Vegetable Slice |
| **Fruit** | 2 pieces fruit | 2 pieces fruit | 2 pieces fruit | 2 pieces fruit | 2 pieces fruit | 2 pieces fruit | 2 pieces fruit |
| **Snack bar** | Snack Bar-Hit100 | Snack Bar-Hit100 | Snack Bar-Nood Bar | Snack Bar-Nood | Snack Bar-Hit100 | Snack Bar-Nood | Snack Bar-Nood |
| **Extra meal** | Oat & berry pikelets | Brown rice & quinoa salad | Cracker snack | Oat & berry pikelets | Cracker snack | Brown rice & quinoa salad | Cracker snack |
| **Lunch** | Bolognese | Chicken/Tofu tikka masala | Pumpkin and mushroom arancini | Pumpkin ravioli | Chicken/Tofu yellow curry | Bolognese | Spanish couscous |
| **Dinner** | Lamb/Bean sweet potato casserole | Crumbed fish and salsa verde | Casserole | Roast lamb with gravy | Salmon pasta bake | Lamb/Bean sweet potato casserole | Cottage pie |

Cells highlighted in orange indicate dishes that remained consistent across all diet groups, while green boxes represent dishes specifically manipulated for each dietary group. Additionally, blue boxes highlight dishes that were manipulated exclusively for high carbohydrate dietary groups.

| **Appendix 3** – Comparison between study completers and non-completers | | | | | |
| --- | --- | --- | --- | --- | --- |
|  | **Completers [n=113]** | **Non-completers [n=15]** | **95% CI** |  | **P value** |
| **Baseline characteristics** |  |  |  | |  |
| Age, years | 69.4 | 68.7 | -2.89 | | 0.31 |
| BMI, kg/m^2^ | 27.5 | 26.9 | -1.76 - 2.95 | | 0.6 |
| PASE, points | 125.8 | 131.6 | -45.1 | | 0.73 |
| Females, n (%) | 75 (66) | 8 (53) | - | | 0.48 |
| Married/de facto, n (%) | 52 (46) | 8 (53) | - | | 0.8 |
| High education, n (%) | 51 (46) | 7 (54) | - | | 0.8 |
| Age pension only, n (%) | 20 (18) | 3 (20) | - | | 1 |
| Ex-smoker, n (%) | 68 (61) | 8 (62) | - | | 1 |
| Australian-born, n (%) | 59 (53) | 4 (31) | - | | 0.23 |
| Excellent/good/fair SRH, n (%) | 107 (98) | 13 (100) | - | | 1 |

BMI, body mass index; PASE, physical activity scale for the elderly; SRH, self-rated health.

| **Appendix 4** **-** Median appetite and palatability scores by diet | | | | | |  | |  | |  | |  |
| --- | --- | --- | --- | --- | --- | --- | --- | --- | --- | --- | --- | --- |
|  | **Median (IQR)** | | | | | | **P value** | | | | | |
| **Appetite score** | **Overall** | **OHF** | **OHC** | **VHF** | **VHC** | | **Protein** | | **F:C ratio** | | **Interaction** | |
| Hunger | 3.5 (5.0) | 3.0 (4.5) | 3.0 (4.5) | 3.5 (5.5) | 3.5 (5.0) | | 0.39 | | 0.38 | | 0.89 | |
| Fullness | 5.0 (4.5) | 5.0 (4.6) | 5.0 (4.5) | 5.0 (4.1) | 5.0 (4.5) | | 0.77 | | 0.48 | | 0.36 | |
| Desire to eat | 3.5 (5.0) | 3.5 (5.0) | 3.5 (5.0) | 3.5 (5.0) | 3.5 (4.5) | | 0.48 | | 0.28 | | 0.77 | |
| Amount could consume | 3.5 (4.0) | 3.5 (4.5) | 3.5 (4.0) | 3.5 (4.5) | 3.5 (4.0) | | 0.6 | | 0.4 | | 0.81 | |
| Desire to eat sweet food | 1.0 (3.0) | 1.0 (3.0) | 1.0 (2.0) | 1.0 (2.5) | 1.5 (3.5) | | 0.4 | | 0.43 | | 0.09 | |
| Desire to eat savoury food | 2.0 (4.5) | 3.5 (4.5) | 1.5 (3.5) | 1.5 (4.5) | 1.5 (4.5) | | <0.001 | | <0.001 | | <0.001 | |
| Contentment | 8.0 (4.0) | 8.5 (4.5) | 8.0 (3.5) | 7.5 (4.0) | 8.0 (3.5) | | 0.31 | | 0.86 | | 0.19 | |
| Irritability | 0.5 (1.0) | 0.5 (1.5) | 0.5 (0.5) | 0.5 (1.5) | 0.5 (1.0) | | 0.81 | | 0.01 | | 0.69 | |
| Depression | 0.5 (0.5) | 0.5 (1.0) | 0.5 (0.5) | 0.5 (0.5) | 0.5 (0.5) | | 0.33 | | 0.27 | | 0.96 | |
| Mental alertness | 9.0 (2.5) | 9.0 (3.0) | 9.0 (2.0) | 8.5 (3.0) | 9.0 (2.0) | | 0.12 | | 0.32 | | 0.12 | |
| **Palatability score** |  |  |  |  |  | |  | |  | |  | |
| Sweet | 4.5 (1.9) | 4.2 (2.1) | 4.4 (1.6) | 4.7 (2.0) | 4.5(1.8) | | 0.21 | | 0.99 | | 0.55 | |
| Savoury | 3.3 (2.0) | 3.5 (2.0) | 3.3 (2.1) | 3.1 (1.7) | 3.3 (2.5) | | 0.2 | | 0.4 | | 0.28 | |
| Tasty | 6.4 (2.1) | 6.5 (2.3) | 6.6 (1.8) | 6.2 (2.1) | 6.2 (2.9) | | 0.99 | | 0.64 | | 0.32 | |
| Pleasant | 6.5 (2.1) | 6.5 (2.2) | 6.6 (1.6) | 6.2 (2.4) | 6.4 (2.9) | | 0.94 | | 0.67 | | 0.35 | |
| Filling | 5.9 (2.3) | 5.8 (2.3) | 6.1 (2.4) | 5.9 (1.8) | 5.9 (2.7) | | 0.73 | | 0.41 | | 0.67 | |
| Satisfying | 6.3 (2.1) | 6.1 (2.1) | 6.4 (1.8) | 5.9 (2.0) | 6.2 (2.8) | | 0.48 | | 0.19 | | 0.15 | |
| Enjoyable | 6.5 (2.3) | 6.3 (2.6) | 6.9 (1.7) | 6.5 (2.4) | 6.4 (3.0) | | 0.39 | | 0.78 | | 0.99 | |
| Prospective food intake | 2.6 (2.8) | 2.9 (2.8) | 2.4 (3.0) | 2.5 (2.9) | 2.5 (2.5) | | 0.8 | | 0.46 | | 0.23 | |
| Interaction refers to the interaction between protein source (plant versus animal) and carbohydrate/fat content (high/low or low/high).  P values determined by GAMM (beta family, logit-link function). | | | | | |  | |  | |  | |  |

| **Appendix 5 -** ANOVA table showing effects of high fat vs high carbohydrate (HF), effects of protein source (Veg) and their interaction (HF:Veg) on changes various markers of health. Model 1 contains follow-up measurements as outcomes with adjustments for baseline results; model 2 is further adjusted for age, BMI, sex and level of physical activity. | | | | |
| --- | --- | --- | --- | --- |
| **Changes in fasting FGF-21 (pmol/L) levels** | | | | |
| **Model 1** | **Df** | **Sum Sq** | **F value** | **p** |
| HF | 1 | 508.483 | 0.033 | 0.86 |
| Veg | 1 | 12178.825 | 0.786 | 0.38 |
| HF:Veg | 1 | 16.042 | 0.001 | 0.97 |
| **Model 2** |  |  |  |  |
| HF | 1 | 3264.911 | 0.207 | 0.65 |
| Veg | 1 | 2498.273 | 0.159 | 0.69 |
| HF:Veg | 1 | 123.644 | 0.008 | 0.93 |
| **Changes in body weight (kg)** | | | | |
| **Model 1** | **Df** | **Sum Sq** | **F value** | **p** |
| HF | 1 | 0.040 | 0.031 | 0.86 |
| Veg | 1 | 5.014 | 3.809 | **0.05** |
| HF:Veg | 1 | 0.365 | 0.277 | 0.60 |
| **Model 2** |  |  |  |  |
| HF | 1 | 0.138 | 0.117 | 0.73 |
| Veg | 1 | 3.282 | 2.771 | 0.10 |
| HF:Veg | 1 | 0.017 | 0.014 | 0.91 |
| **Changes in body fat mass (kg)** | | | | |
| **Model 1** | **Df** | **Sum Sq** | **F value** | **p** |
| HF | 1 | 0.088 | 0.035 | 0.85 |
| Veg | 1 | 0.691 | 0.273 | 0.60 |
| HF:Veg | 1 | 0.059 | 0.023 | 0.88 |
| **Model 2** |  |  |  |  |
| HF | 1 | 1.434 | 0.532 | 0.47 |
| Veg | 1 | 1.117 | 0.414 | 0.52 |
| HF:Veg | 1 | 0.015 | 0.005 | 0.94 |
| **Changes in body fat mass (%)** | | | | |
| **Model 1** | **Df** | **Sum Sq** | **F value** | **p** |
| HF | 1 | 0.237 | 0.060 | 0.81 |
| Veg | 1 | 0.348 | 0.088 | 0.77 |
| HF:Veg | 1 | 0.158 | 0.040 | 0.84 |
| **Model 2** |  |  |  |  |
| HF | 1 | 2.228 | 0.525 | 0.47 |
| Veg | 1 | 0.009 | 0.002 | 0.96 |
| HF:Veg | 1 | 0.023 | 0.048 | 0.83 |
| **Changes in body fat free mass (kg)** | | | | |
| **Model 1** | **Df** | **Sum Sq** | **F value** | **p** |
| HF | 1 | 0.452 | 0.275 | 0.60 |
| Veg | 1 | 1.465 | 0.892 | 0.35 |
| HF:Veg | 1 | 0.245 | 0.149 | 0.70 |
| **Model 2** |  |  |  |  |
| HF | 1 | 0.901 | 0.508 | 0.48 |
| Veg | 1 | 1.671 | 0.942 | 0.33 |
| HF:Veg | 1 | 0.463 | 0.261 | 0.61 |
| **Changes in body fat free mass (%)** | | | | |
| **Model 1** | **Df** | **Sum Sq** | **F value** | **p** |
| HF | 1 | 0.361 | 0.091 | 0.76 |
| Veg | 1 | 0.355 | 0.090 | 0.77 |
| HF:Veg | 1 | 0.099 | 0.025 | 0.87 |
| **Model 2** |  |  |  |  |
| HF | 1 | 2.628 | 0.621 | 0.43 |
| Veg | 1 | 0.008 | 0.002 | 0.97 |
| HF:Veg | 1 | 0.308 | 0.073 | 0.79 |
| **Changes in waist circumference (cm)** | | | | |
| **Model 1** | **Df** | **Sum Sq** | **F value** | **p** |
| HF | 1 | 9.935 | 0.095 | 0.76 |
| Veg | 1 | 2.444 | 0.023 | 0.88 |
| HF:Veg | 1 | 8.368 | 0.080 | 0.78 |
| **Model 2** |  |  |  |  |
| HF | 1 | 35.045 | 0.325 | 0.57 |
| Veg | 1 | 7.673 | 0.071 | 0.79 |
| HF:Veg | 1 | 7.519 | 0.070 | 0.79 |
| **Changes in walk speed (m/sec)** | | | | |
| **Model 1** | **Df** | **Sum Sq** | **F value** | **p** |
| HF | 1 | 0.056 | 0.093 | 0.76 |
| Veg | 1 | 0.463 | 0.771 | 0.38 |
| HF:Veg | 1 | 0.083 | 0.138 | 0.71 |
| **Model 2** |  |  |  |  |
| HF | 1 | 0.047 | 0.088 | 0.77 |
| Veg | 1 | 0.121 | 0.229 | 0.63 |
| HF:Veg | 1 | 0.002 | 0.004 | 0.95 |
| **Changes in chair stand test time (sec)** | | | | |
| **Model 1** | **Df** | **Sum Sq** | **F value** | **p** |
| HF | 1 | 0.392 | 0.104 | 0.75 |
| Veg | 1 | 2.363 | 0.629 | 0.43 |
| HF:Veg | 1 | 0.007 | 0.002 | 0.96 |
| **Model 2** |  |  |  |  |
| HF | 1 | 2.421 | 0.747 | 0.39 |
| Veg | 1 | 2.319 | 0.716 | 0.40 |
| HF:Veg | 1 | 1.039 | 168.526 | 0.57 |
| **Changes in grip strength (kg)** | | | | |
| **Model 1** | **Df** | **Sum Sq** | **F value** | **p** |
| HF | 1 | 2.139 | 0.183 | 0.70 |
| Veg | 1 | 0.852 | 0.759 | 0.39 |
| HF:Veg | 1 | 12.136 | 1.041 | 0.31 |
| **Model 2** |  |  |  |  |
| HF | 1 | 6.461 | 0.530 | 0.47 |
| Veg | 1 | 17.664 | 1.448 | 0.23 |
| HF:Veg | 1 | 23.282 | 1.908 | 0.17 |
| **Changes in SBP (mmHg)** | | | | |
| **Model 1** | **Df** | **Sum Sq** | **F value** | **p** |
| HF | 1 | 426.485 | 1.884 | 0.17 |
| Veg | 1 | 755.974 | 3.339 | 0.07 |
| HF:Veg | 1 | 672.498 | 2.970 | 0.09 |
| **Model 2** |  | | | |
| HF | 1 | 242.955 | 1.071 | 0.30 |
| Veg | 1 | 386.728 | 1.705 | 0.20 |
| HF:Veg | 1 | 498.902 | 2.200 | 0.14 |
| **Changes in DBP (mmHg)** | | | | |
| **Model 1** | **Df** | **Sum Sq** | **F value** | **p** |
| HF | 1 | 3.834 | 0.135 | 0.71 |
| Veg | 1 | 139.144 | 4.912 | **0.03** |
| HF:Veg | 1 | 1.836 | 0.065 | 0.80 |
| **Model 2** |  |  |  |  |
| HF | 1 | 7.447 | 0.270 | 0.61 |
| Veg | 1 | 117.839 | 4.265 | **0.04** |
| HF:Veg | 1 | 6.883 | 0.249 | 0.62 |
| **Changes in HOMA-IR (mmHg)** | | | | |
| **Model 1** | **Df** | **Sum Sq** | **F value** | **p** |
| HF | 1 | 0.109 | 1.313 | 0.25 |
| Veg | 1 | 0.029 | 0.344 | 0.56 |
| HF:Veg | 1 | 0.181 | 2.185 | 0.14 |
| **Model 2** |  |  |  |  |
| HF | 1 | 0.070 | 0.906 | 0.34 |
| Veg | 1 | 0.024 | 0.318 | 0.57 |
| HF:Veg | 1 | 0.125 | 1.629 | 0.21 |
| **Changes in glucose (mmol/L)** | | | | |
| **Model 1** | **Df** | **Sum Sq** | **F value** | **p** |
| HF | 1 | 2E-06 | 9E-06 | 1.00 |
| Veg | 1 | 0.896 | 4.959 | **0.03** |
| HF:Veg | 1 | 0.094 | 0.518 | 0.47 |
| **Model 2** |  |  |  |  |
| HF | 1 | 0.147 | 0.822 | 0.37 |
| Veg | 1 | 0.322 | 1.795 | 0.18 |
| HF:Veg | 1 | 0.001 | 0.004 | 0.95 |
| **Changes in insulin (pmol/L)** | | | | |
| **Model 1** | **Df** | **Sum Sq** | **F value** | **p** |
| HF | 1 | 293.511 | 1.296 | 0.26 |
| Veg | 1 | 56.649 | 0.250 | 0.62 |
| HF:Veg | 1 | 519.212 | 2.292 | 0.13 |
| **Model 2** |  |  |  |  |
| HF | 1 | 180.738 | 0.874 | 0.35 |
| Veg | 1 | 50.197 | 0.243 | 0.62 |
| HF:Veg | 1 | 355.996 | 1.721 | 0.19 |
| **Changes in total cholesterol (mmol/L)** | | | | |
| **Model 1** | **Df** | **Sum Sq** | **F value** | **p** |
| HF | 1 | 0.130 | 0.234 | 0.63 |
| Veg | 1 | 2.689 | 4.836 | **0.03** |
| HF:Veg | 1 | 0.371 | 0.667 | 0.42 |
| **Model 2** |  |  |  |  |
| HF | 1 | 0.087 | 0.138 | 0.71 |
| Veg | 1 | 1.750 | 2.786 | 0.10 |
| HF:Veg | 1 | 0.343 | 0.546 | 0.46 |
| **Changes in HDL cholesterol (mmol/L)** | | | | |
| **Model 1** | **Df** | **Sum Sq** | **F value** | **p** |
| HF | 1 | 0.043 | 0.968 | 0.33 |
| Veg | 1 | 0.002 | 0.051 | 0.82 |
| HF:Veg | 1 | 0.004 | 0.081 | 0.78 |
| **Model 2** |  |  |  |  |
| HF | 1 | 0.012 | 0.256 | 0.61 |
| Veg | 1 | 0.015 | 0.306 | 0.58 |
| HF:Veg | 1 | 0.001 | 0.020 | 0.89 |
| **Changes in LDL cholesterol (mmol/L)** | | | | |
| **Model 1** | **Df** | **Sum Sq** | **F value** | **p** |
| HF | 1 | 0.000 | 0.000 | 0.99 |
| Veg | 1 | 1.416 | 3.278 | 0.07 |
| HF:Veg | 1 | 0.182 | 0.420 | 0.52 |
| **Model 2** |  |  |  |  |
| HF | 1 | 0.043 | 0.086 | 0.77 |
| Veg | 1 | 0.972 | 1.925 | 0.17 |
| HF:Veg | 1 | 0.249 | 0.493 | 0.48 |
| **Changes in triglycerides (mmol/L)** | | | | |
| **Model 1** | **Df** | **Sum Sq** | **F value** | **p** |
| HF | 1 | 0.064 | 0.335 | 0.56 |
| Veg | 1 | 0.010 | 0.054 | 0.82 |
| HF:Veg | 1 | 0.030 | 0.156 | 0.69 |
| **Model 2** |  |  |  |  |
| HF | 1 | 0.304 | 1.870 | 0.18 |
| Veg | 1 | 0.069 | 0.425 | 0.52 |
| HF:Veg | 1 | 0.118 | 0.726 | 0.40 |
| **Changes in diversity observed** | | | | |
| \| **Model 1** \| **Df** \| **Sum Sq** \| **F value** \| **p** \| \| --- \| --- \| --- \| --- \| --- \| \| HF \| 1 \| 3.537 \| 0.004 \| 0.95 \| \| Veg \| 1 \| 942.553 \| 1.024 \| 0.31 \| \| HF:Veg \| 1 \| 4099.698 \| 4.452 \| 0.04 \| \| **Model 2** \|  \|  \|  \|  \| \| HF \| 1 \| 249.109 \| 0.288 \| 0.59 \| \| Veg \| 1 \| 787.744 \| 0.912 \| 0.34 \| \| HF:Veg \| 1 \| 4457.661 \| 5.162 \| 0.03 \| | | | | |
| **Changes in diversity Shannon** | | | | |
| \| **Model 1** \| **Df** \| **Sum Sq** \| **F value** \| **p** \| \| --- \| --- \| --- \| --- \| --- \| \| HF \| 1 \| 0.015 \| 0.100 \| 0.75 \| \| Veg \| 1 \| 0.192 \| 1.310 \| 0.26 \| \| HF:Veg \| 1 \| 1.027 \| 7.022 \| 0.01 \| \| **Model 2** \|  \|  \|  \|  \| \| HF \| 1 \| 0.001 \| 0.006 \| 0.94 \| \| Veg \| 1 \| 0.105 \| 0.752 \| 0.39 \| \| HF:Veg \| 1 \| 0.969 \| 6.915 \| 0.01 \| | | | | |
| **Changes in diversity InvSimpson** | | | | |
| \| **Model 1** \| **Df** \| **Sum Sq** \| **F value** \| **p** \| \| --- \| --- \| --- \| --- \| --- \| \| HF \| 1 \| 4.806 \| 0.033 \| 0.86 \| \| Veg \| 1 \| 85.387 \| 0.585 \| 0.45 \| \| HF:Veg \| 1 \| 1278.946 \| 8.758 \| 0.004 \| \| **Model 2** \|  \|  \|  \|  \| \| HF \| 1 \| 7.558 \| 0.052 \| 0.82 \| \| Veg \| 1 \| 36.348 \| 0.252 \| 0.62 \| \| HF:Veg \| 1 \| 1271.626 \| 8.831 \| 0.004 \| | | | | |
| **Changes in diversity Fisher** | | | | |
| \| **Model 1** \| **Df** \| **Sum Sq** \| **F value** \| **p** \| \| --- \| --- \| --- \| --- \| --- \| \| HF \| 1 \| 0.001 \| 0.00 \| 0.99 \| \| Veg \| 1 \| 22.816 \| 1.175 \| 0.28 \| \| HF:Veg \| 1 \| 87.203 \| 4.490 \| 0.04 \| \| **Model 2** \|  \|  \|  \|  \| \| HF \| 1 \| 5.962 \| 0.327 \| 0.57 \| \| Veg \| 1 \| 17.932 \| 0.985 \| 0.32 \| \| HF:Veg \| 1 \| 97.943 \| 5.380 \| 0.02 \| | | | | |
| **Changes in diversity evenness** | | | | |
| \| **Model 1** \| **Df** \| **Sum Sq** \| **F value** \| **p** \| \| --- \| --- \| --- \| --- \| --- \| \| HF \| 1 \| 0.000 \| 0.091 \| 0.76 \| \| Veg \| 1 \| 0.003 \| 1.798 \| 0.18 \| \| HF:Veg \| 1 \| 0.009 \| 6.620 \| 0.01 \| \| **Model 2** \|  \|  \|  \|  \| \| HF \| 1 \| 0.000 \| 0.006 \| 0.94 \| \| Veg \| 1 \| 0.001 \| 0.983 \| 0.32 \| \| HF:Veg \| 1 \| 0.009 \| 6.683 \| 0.01 \| | | | | |
| **Changes in *firmicutes* level** | | | | |
| **Model 1** | **Df** | **Sum Sq** | **F value** | **p** |
| HF | 1 | 93.546 | 0.794 | 0.38 |
| Veg | 1 | 0.535 | 0.005 | 0.95 |
| HF:Veg | 1 | 316.394 | 2.687 | 0.10 |
| **Model 2** |  |  |  |  |
| HF | 1 | 110.597 | 0.838 | 0.36 |
| Veg | 1 | 19.911 | 0.151 | 0.70 |
| HF:Veg | 1 | 223.206 | 1.692 | 0.20 |
| **Changes in *bacteroidetes* level** | | | | |
| **Model 1** | **Df** | **Sum Sq** | **F value** | **p** |
| HF | 1 | 7.232 | 0.151 | 0.70 |
| Veg | 1 | 77.384 | 1.616 | 0.21 |
| HF:Veg | 1 | 30.981 | 0.647 | 0.42 |
| **Model 2** |  |  |  |  |
| HF | 1 | 48.946 | 1.120 | 0.29 |
| Veg | 1 | 32.013 | 0.733 | 0.39 |
| HF:Veg | 1 | 0.006 | 0.000 | 0.99 |
| **Changes in *actinobacteria* level** | | | | |
| **Model 1** | **Df** | **Sum Sq** | **F value** | **p** |
| HF | 1 | 45.410 | 0.888 | 0.35 |
| Veg | 1 | 213.684 | 4.180 | **0.04** |
| HF:Veg | 1 | 4.629 | 0.091 | 0.76 |
| **Model 2** |  |  |  |  |
| HF | 1 | 17.848 | 0.291 | 0.59 |
| Veg | 1 | 200.781 | 3.274 | 0.07 |
| HF:Veg | 1 | 3.091 | 0.050 | 0.82 |
| **Changes in *verrucomicrobia* level** | | | | |
| **Model 1** | **Df** | **Sum Sq** | **F value** | **p** |
| HF | 1 | 26.059 | 0.813 | 0.37 |
| Veg | 1 | 1.218 | 0.038 | 0.85 |
| HF:Veg | 1 | 7.974 | 0.249 | 0.62 |
| **Model 2** |  |  |  |  |
| HF | 1 | 21.110 | 0.677 | 0.41 |
| Veg | 1 | 7.442 | 0.239 | 0.63 |
| HF:Veg | 1 | 19.685 | 0.631 | 0.43 |
| **Changes in *euryarchaeota* level** | | | | |
| **Model 1** | **Df** | **Sum Sq** | **F value** | **p** |
| HF | 1 | 9.234 | 2.069 | 0.15 |
| Veg | 1 | 11.903 | 2.667 | 0.11 |
| HF:Veg | 1 | 0.037 | 0.008 | 0.93 |
| **Model 2** |  |  |  |  |
| HF | 1 | 6.956 | 1.610 | 0.21 |
| Veg | 1 | 10.023 | 2.320 | 0.13 |
| HF:Veg | 1 | 0.153 | 0.035 | 0.85 |
| **Changes in *synergistetes* level** | | | | |
| **Model 1** | **Df** | **Sum Sq** | **F value** | **p** |
| HF | 1 | 0.012 | 2.496 | 0.12 |
| Veg | 1 | 0.001 | 0.116 | 0.73 |
| HF:Veg | 1 | 0.000 | 0.067 | 0.80 |
| **Model 2** |  |  |  |  |
| HF | 1 | 0.016 | 2.688 | 0.11 |
| Veg | 1 | 0.003 | 0.485 | 0.49 |
| HF:Veg | 1 | 0.002 | 0.300 | 0.59 |
| **Changes in *proteobacteria* level** | | | | |
| **Model 1** | **Df** | **Sum Sq** | **F value** | **p** |
| HF | 1 | 4.296 | 0.375 | 0.54 |
| Veg | 1 | 20.710 | 1.807 | 0.18 |
| HF:Veg | 1 | 42.119 | 3.674 | 0.06 |
| **Model 2** |  |  |  |  |
| HF | 1 | 8.741 | 0.699 | 0.41 |
| Veg | 1 | 15.725 | 1.258 | 0.27 |
| HF:Veg | 1 | 65.091 | 5.208 | **0.03** |
| **Changes in acetate level** | | | | |
| **Model 1** | **Df** | **Sum Sq** | **F value** | **p** |
| HF | 1 | 0.013 | 0.000 | 1.00 |
| Veg | 1 | 1625.052 | 2.274 | 0.14 |
| HF:Veg | 1 | 1639.193 | 2.294 | 0.13 |
| **Model 2** |  |  |  |  |
| HF | 1 | 44.254 | 0.059 | 0.81 |
| Veg | 1 | 1312.459 | 1.736 | 0.19 |
| HF:Veg | 1 | 2864.399 | 3.789 | 0.06 |

DBP, diastolic blood pressure; BMI, body mass index; HOMA-IR, homeostatic model assessment for insulin resistance

**Appendix 6 - Predictors of changes in muscle strength;** Cumming plots showing changes in chair stand test (A), grip strength (B) and walking speed (C) by diet. Error bars are 95% confidence intervals found by bootstrapping. BL, baseline assessment, FN, final assessment. OHF, omnivorous high fat; OHC, omnivorous high carbohydrate; VHF, semi-vegetarian high fat; VHC, semi-vegetarian high carbohydrate.


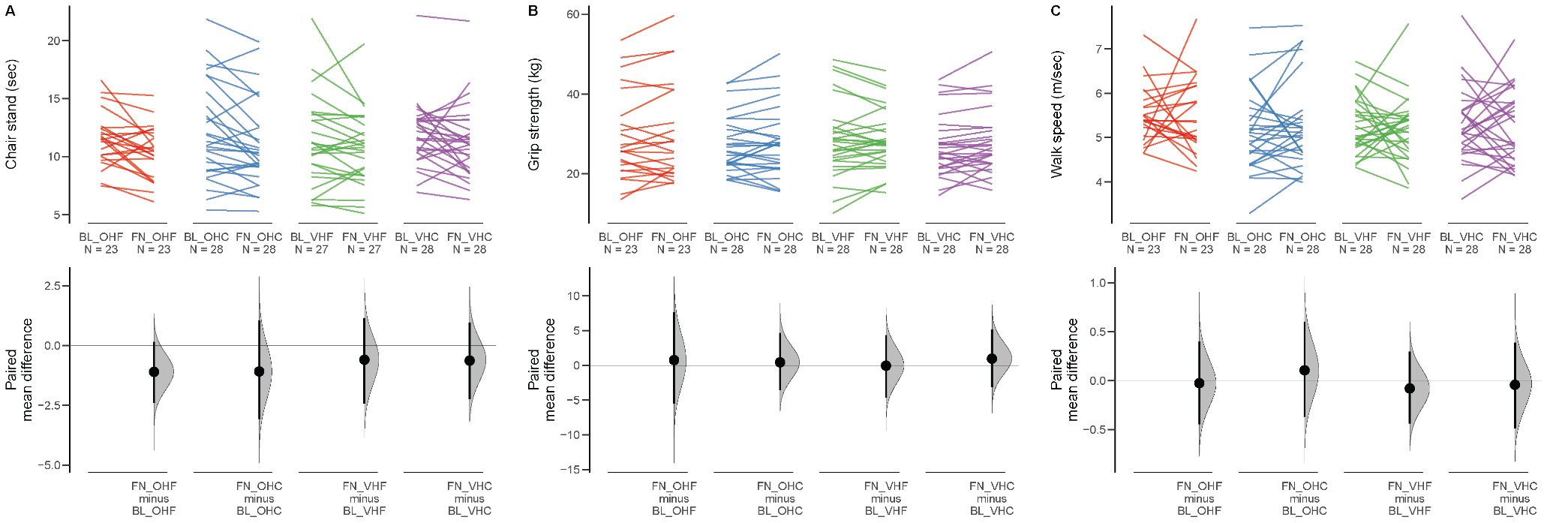


**Appendix 7 - Predictors of change in cardiometabolic health;** Cumming plots showing changes in systolic blood pressure [SBP] (A), high-density lipoprotein cholesterol [HLDc] (B), low-density lipoprotein cholesterol [LDLc] (C), triglycerides (D), insulin (E) and Homeostatic Model Assessment for Insulin Resistance [HOMA IR](F) by diet. Error bars are 95% confidence intervals found by bootstrapping. BL, baseline assessment; FN, final assessment. OHF, omnivorous high fat; OHC, omnivorous high carbohydrate; VHF, semi-vegetarian high fat; VHC, semi-vegetarian high carbohydrate.

**C**

**B**


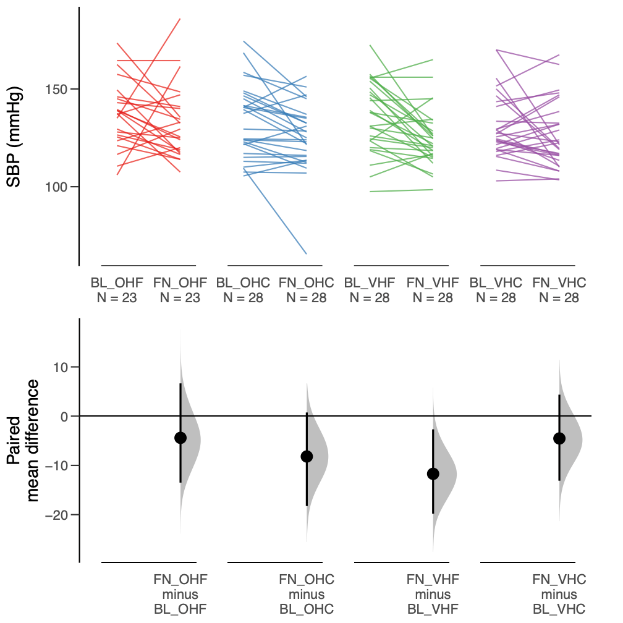

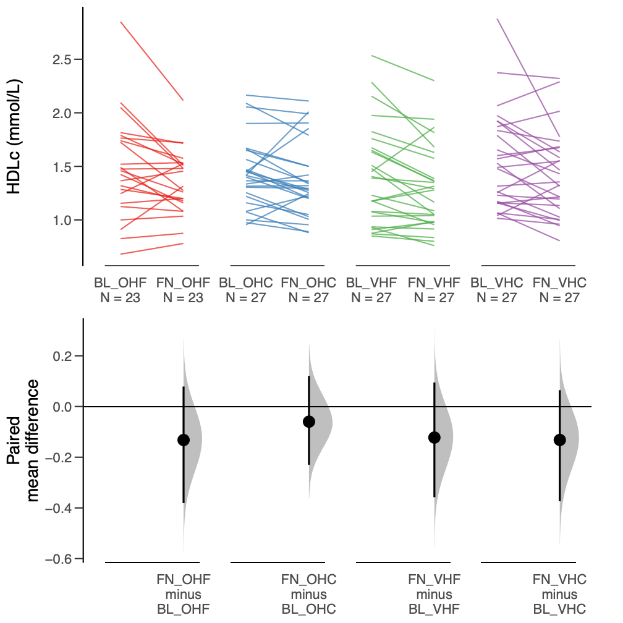

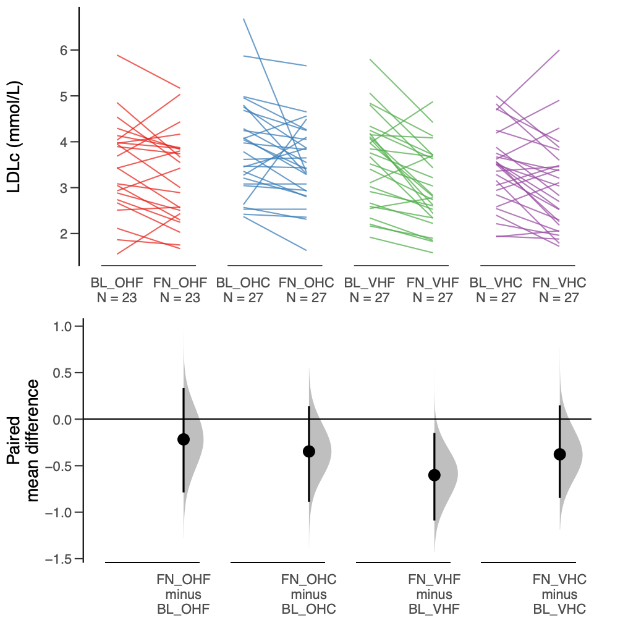


**A**


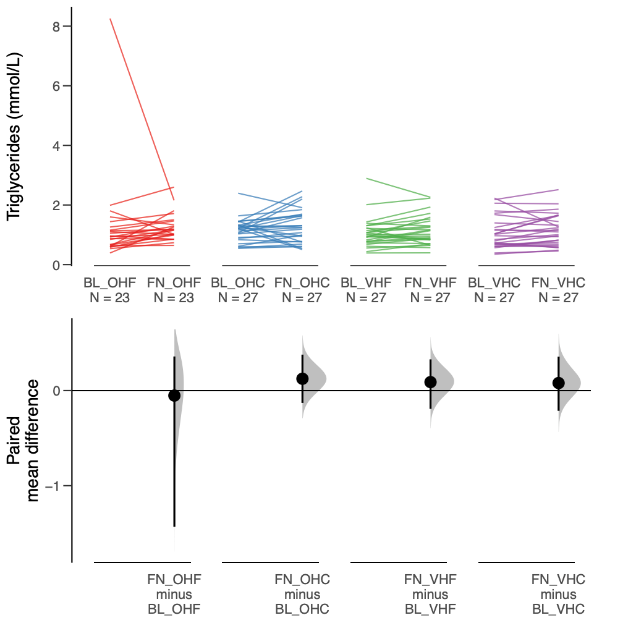

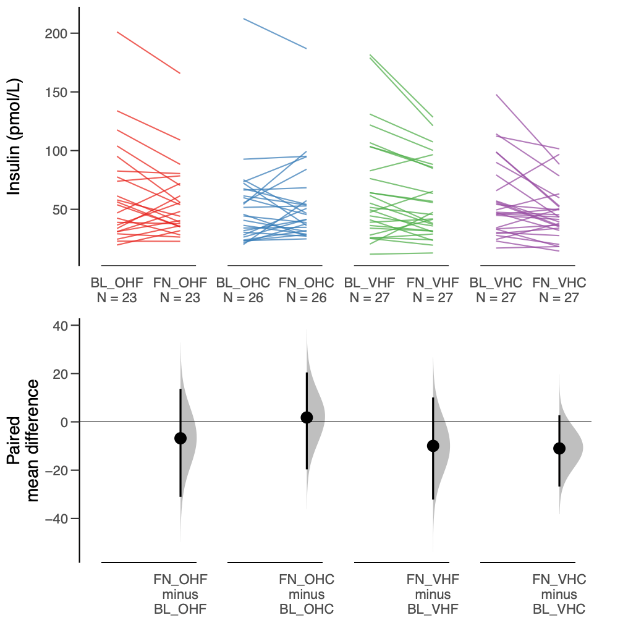

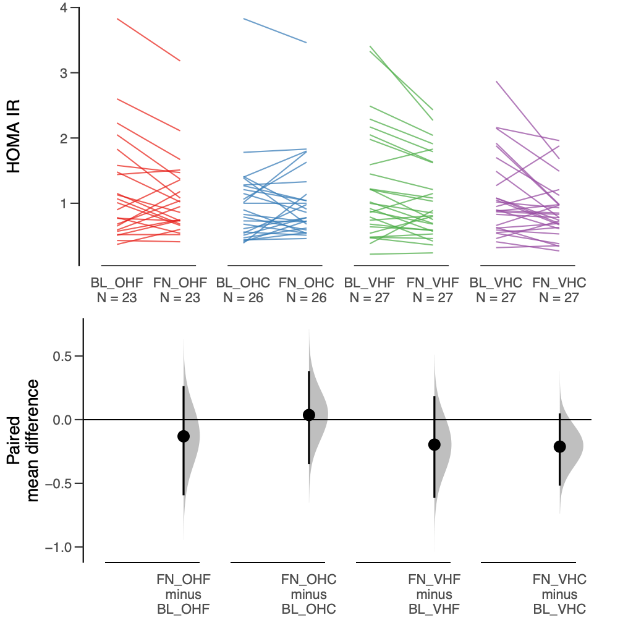


**F**

**E**

**D**

**Appendix 8 - Predictors of changes of gut microbiota;** Heatmap showing correlation between changes in short-chain fatty acids (SCFA), changes in gut microbiome diversity (A) and changes in abundance of bacterial phila (B) with dietary changes, changes in HOMA IR, BCAA levels and levels of physical activity. Changes in all measures of gut microbiome diversity were strongly correlated with changes in body weight and dietary intake. FN, final assessment. OHF, omnivorous high fat; OHC, omnivorous high carbohydrate; VHF, semi-vegetarian high fat; VHC, semi-vegetarian high carbohydrate.

**A**

**B**


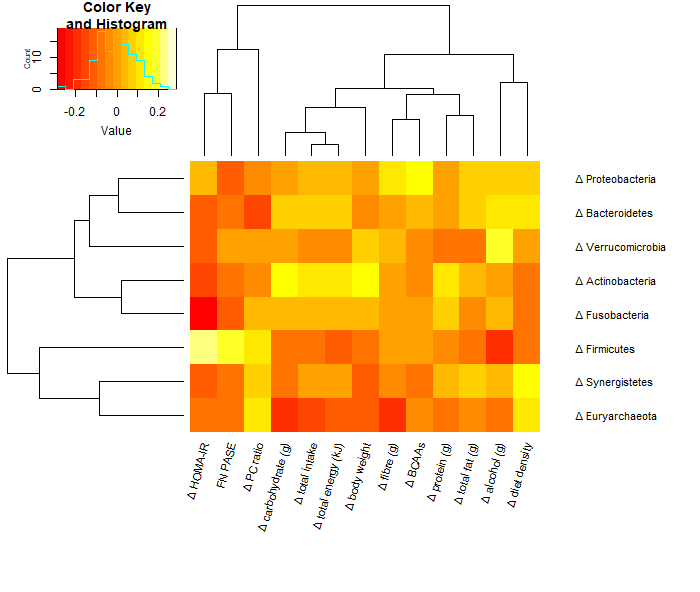


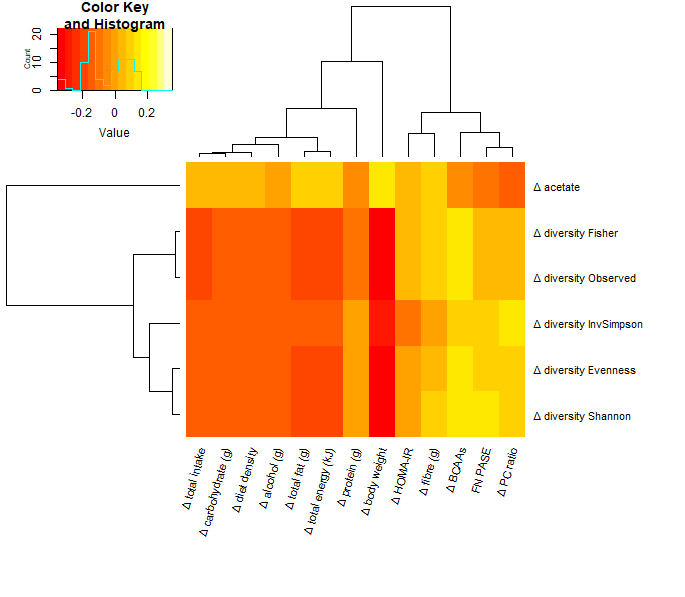


**Appendix 9 -** Cumming plots showing changes in diversity observed (A), diversity Shannon (B), diversity InvSimpson (C), diversity Fisher (D), diversity evenness (E), firmicutes (F), bacteroidetes (G), actinobacteria (H), verrucomicrobia (I), euryachaeota (J), synergistetes (K), proteobacteria (L) and acetate (M). BL, baseline assessment; FN, final assessment. OHF, omnivorous high fat; OHC, omnivorous high carbohydrate; VHF, semi-vegetarian high fat; VHC, semi-vegetarian high carbohydrate.


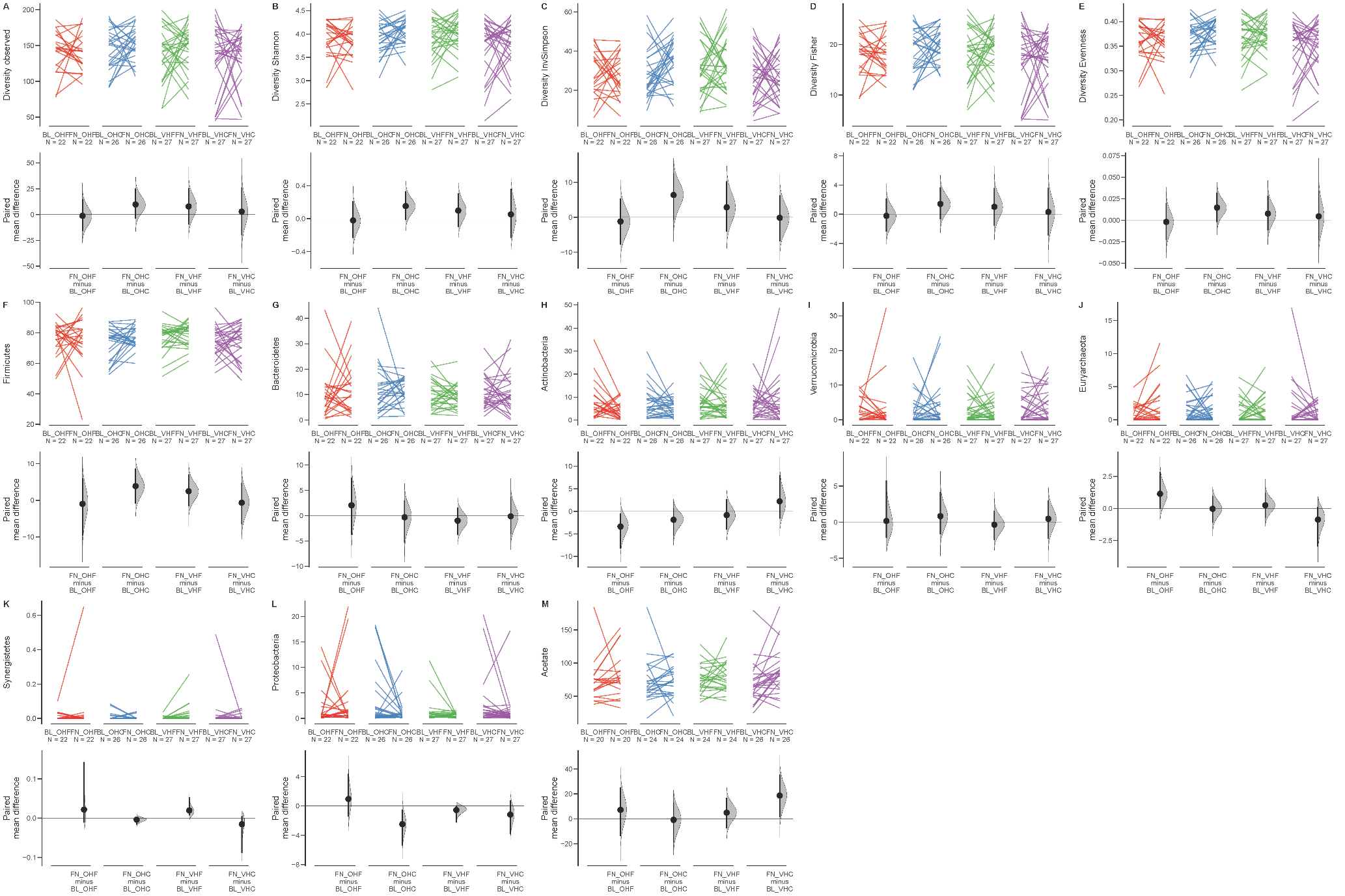


| **Appendix 10 -** Overall comparison of baseline vs final measures (all diets pooled), and 2x2 effects of protein source and fat/carbohydrate ratio on change of metabolites levels from baseline. | | | | | | | | |
| --- | --- | --- | --- | --- | --- | --- | --- | --- |
| **Metabolomics** | **Baseline** | **Final** | **Mean difference** | **% difference** | **p value** | **Protein source** | **Fat/carbohydrate ratio** | **Interaction** |
| BCAA | 1 | 0.99 | -0.01 | -0.51 | **0.01** | 0.96[0.98] | 0.98[0.73] | 0.82[0.73] |
| Alanine | 1 | 1.01 | 0.01 | 1.2 | 0.25 | 0.73[0.85] | 0.19[0.12] | 0.26[0.20] |
| Serine | 1 | 1.01 | 0.01 | 0.5 | 0.43 | 0.49[0.27] | 0.59[0.23] | 0.17[0.31] |
| Proline | 1 | 1 | 0 | -0.03 | 0.98 | 0.61[0.43] | 0.97[1.00] | 0.13[0.18] |
| Valine | 1 | 0.99 | -0.01 | -0.7 | **<0.001** | 0.37[0.44] | 1.00[0.71] | 0.14[0.08] |
| Threonine | 1 | 1 | 0 | 0.1 | 0.69 | **0.04**[0.08] | 0.25[0.18] | 0.40[0.89] |
| Cysteine | 1 | 1.01 | 0.01 | 1.1 | 0.3 | 0.78[0.36] | 0.12[0.17] | 0.16[0.28] |
| Isoleucine | 1 | 1 | 0 | -0.4 | 0.27 | 0.97[0.72] | 0.76[0.66] | 0.79[0.24] |
| Leucine | 1 | 1 | 0 | -0.4 | 0.19 | 0.63[0.94] | 0.78[0.92] | 0.39[0.46] |
| Aspartate | 1 | 1.03 | 0.03 | 3.2 | **<0.001** | **0.01**[**0.01**] | 0.75[0.56] | 0.85[0.85] |
| Lysine | 1 | 1 | 0 | 0.4 | 0.02 | 0.59[0.48] | 0.88[0.86] | 0.95[0.94] |
| Glutamine | 1 | 1.01 | 0.01 | 1 | **<0.001** | 0.34[0.27] | 0.12[0.16] | 0.72[0.52] |
| Glutamate | 1 | 1 | 0 | 0.3 | 0.49 | 0.20[0.05] | 0.94[0.62] | 0.08[0.10] |
| Methionine | 1 | 1 | 0 | 0.3 | 0.42 | 0.62[0.76] | 0.64[0.95] | 0.42[0.54] |
| Glucose | 1 | 0.99 | -0.01 | -0.6 | 0.25 | 0.76[0.82] | 0.23[0.23] | 0.47[0.82] |
| Phenylalanine | 1 | 1 | 0 | -0.2 | 0.25 | 0.11[0.24] | 0.08[0.21] | 0.65[0.96] |
| Arginine | 1 | 1 | 0 | -0.07 | 0.76 | 0.06[0.35] | 0.63[0.95] | 0.82[0.88] |
| Tyrosine | 1 | 1 | 0 | -0.46 | 0.06 | 0.95[0.56] | 0.73[0.57] | 0.33[0.78] |
| Methionine sulfone | 1 | 1 | 0 | -0.45 | 0.1 | 0.98[0.64] | 0.63[0.93] | 0.70[0.88] |
| Tryptophan | 1 | 1 | 0 | -0.01 | 0.98 | 0.89[0.75] | 0.54[0.32] | 0.98[0.46] |
| Asparagine | 1 | 1.+02 | 0.02 | 1.55 | 0.2 | 0.92[0.98] | 0.83[0.79] | 0.29[0.12] |
| 1-methylhistamine | 1 | 1 | 0 | 0.27 | 0.75 | 0.31[0.36] | 0.73[0.80] | 0.88[0.82] |
| 2'-deoxyadenosine | 1 | 1 | 0 | 0 | 1 | 0.58[0.99] | 0.38[0.35] | 0.70[0.72] |
| 2'-deoxycytidine | 1 | 1.01 | 0.01 | 1.48 | 0.19 | 0.39[0.36] | 0.63[0.87] | 0.66[0.25] |
| 3-hydroxykynurenine | 1 | 1.01 | 0.01 | 0.85 | 0.49 | 0.98[0.69] | 0.56[0.68] | 0.46[0.38] |
| 5-hydroxyindoleacetic acid | 1 | 1.01 | 0.01 | 0.73 | 0.2 | 0.40[0.21] | 0.69[0.67] | 0.93[0.49] |
| Adenosine | 1 | 1.03 | 0.03 | 2.71 | 0.03 | **0.05[0.04**] | 0.81[0.83] | 0.61[0.47] |
| Asymmetric dimethylarginine | 1 | 1 | 0 | 0.37 | 0.46 | 0.62[0.99] | 0.26[0.34] | 0.68[0.84] |
| α-keto-δ-(N(G),N(G)-dimethylguanidino)valeric acid | 1 | 1.01 | 0.01 | 1.07 | 0.25 | 0.20[0.07] | 0.91[0.44] | 0.83[0.85] |
| Anserine | 1 | 0.99 | -0.01 | -0.64 | 0.61 | 0.72[0.87] | 0.44[0.37] | 0.36[0.11] |
| Betaine | 1 | 1 | 0 | 0.1 | 0.71 | 0.38[0.34] | 1.00[0.91] | 0.97[0.69] |
| Butyryl carnitine | 1 | 0.99 | -0.01 | -1.09 | **<0.001** | 0.85[0.46] | **0.03**[0.15] | 0.41[0.41] |
| Cyclic adenosine monophosphate | 1 | 1 | 0 | 0.06 | 0.98 | 013[0.09] | 0.97[0.54] | 0.08[0.36] |
| Carnosine | 1 | 1 | 0 | 0.47 | 0.71 | 0.65[0.90] | 0.42[0.49] | 0.45[0.21] |
| Citrulline | 1 | 1.01 | 0.01 | 1.1 | 0.2 | 0.16[**0.01**] | 0.52[0.37] | 0.62[0.41] |
| Creatine | 1 | 1.01 | 0.01 | 1.22 | 0.12 | 0.28[**0.04**] | 0.86[0.47] | 0.70[0.24] |
| Cysteamine | 1 | 0.97 | -0.03 | -2.96 | **<0.001** | 0.84[0.68] | 0.54[0.92] | 0.70[0.92] |
| Cysteamine | 1 | 1.05 | 0.05 | 5.06 | 0.01 | 0.59[0.31] | **0.03**[0.29] | 0.10[0.12] |
| Cytidine | 1 | 1.11 | 0.11 | 11.24 | **<0.001** | **0.002[0.01]** | **<0.001[<0.001]** | 0.27[0.37] |
| Cytosine | 1 | 0.99 | -0.01 | -0.67 | 0.16 | 0.83[0.27] | 0.16[0.14] | 0.32[0.36] |
| Gamma aminobutyric acid | 1 | 0.99 | -0.01 | -0.86 | **0.01** | 0.20[0.22] | 0.58[0.58] | 0.24[0.81] |
| Histamine | 1 | 0.99 | -0.01 | -1.35 | 0.23 | 0.94[0.87] | 0.17[0.42] | 0.82[0.63] |
| Kynurenic acid | 1 | 0.98 | -0.02 | -1.67 | **<0.001** | 0.11[0.13] | 0.66[0.95] | **0.03**[0.27] |
| Ornithine | 1 | 1 | 0 | 0.06 | 0.84 | 0.24[0.15] | 0.13[0.13] | 0.32[0.77] |
| Phosphocholine | 1 | 1 | 0 | 0.32 | 0.53 | 0.24[0.42] | 0.41[0.55] | 0.55[0.44] |
| Serotonin | 1 | 1 | 0 | 0.2 | 0.77 | 0.44[0.81] | 0.07[0.23] | 0.12[0.20] |
| Spermine | 1 | 1 | 0 | -0.21 | 0.71 | 1.00[0.51] | 0.30[0.78] | 0.60[0.73] |
| Taurine | 1 | 1 | 0 | 0.47 | 0.35 | **0.01[0.002]** | 0.23[0.15] | 0.50[0.38] |
| Thiamine | 1 | 0.97 | -0.03 | -2.57 | 0.01 | 0.94[0.55] | 0.10[0.30] | 0.82[0.36] |
| trans-hydroxyproline | 1 | 1 | 0 | 0.2 | 0.79 | 0.67[0.73] | 0.51[0.21] | 0.15[0.55] |
| Triiodothyronine | 1 | 1 | 0 | -0.13 | 0.71 | **0.01**[**0.01**] | 0.45[0.52] | 0.75[0.95] |
| Trimethylamine N-oxide | 1 | 0.98 | -0.02 | -1.82 | 0.2 | 0.87[0.84] | **0.03**[0.16] | 0.90[0.47] |
| Thymidine | 1 | 0.99 | -0.01 | -0.61 | 0.69 | 0.78[0.79] | 0.68[0.87] | 0.11[0.07] |
| Uridine | 1 | 1 | 0 | -0.28 | 0.12 | 0.06[**0.03**] | 0.07[0.10] | 0.12[0.15] |
| Deazaadenosine | 1 | 0.98 | -0.02 | -1.64 | **0** | 0.45[0.60] | 0.31[0.35] | 0.97[0.95] |
| 3-indolepropionic acid | 1 | 1.05 | 0.05 | 4.78 | **<0.001** | **0.01**[**0.01**] | **0.02**[0.08] | 0.28[0.38] |
| Acetyl carnitine | 1 | 1 | 0 | -0.29 | 0.69 | 0.94[0.72] | 0.68[0.34] | 0.69[0.57] |
| Acetylcholine | 1 | 0.99 | -0.01 | -0.87 | 0.34 | 0.38[0.64] | 0.90[0.79] | 0.71[0.85] |
| Carnitine | 1 | 0.99 | -0.01 | -0.55 | 0.02 | 0.20[0.29] | 0.34[0.24] | 0.35[0.79] |
| L-Homoserine | 1 | 1.01 | 0.01 | 1.1 | 0.2 | **0.05**[**0.03**] | 0.14[**0.03**] | 0.64[0.26] |
| N-acetyl glutamine | 1 | 0.99 | -0.01 | -1.27 | **<0.001** | 0.23[0.31] | 0.47[0.65] | 0.44[0.36] |
| NG-monomethyl-L-arginine | 1 | 1.02 | 0.02 | 1.53 | 0.17 | 0.14[0.08] | 0.11[0.25] | 0.69[0.17] |
| Purine | 1 | 1.01 | 0.01 | 0.51 | 0.69 | **0.03**[0.11] | 0.67[0.22] | 0.51[0.89] |
| Pyridoxine | 1 | 0.98 | -0.02 | -2.47 | 0.01 | 0.77[0.68] | 0.70[0.57] | 0.53[0.87] |
| Riboflavin | 1 | 0.99 | -0.01 | -0.64 | 0.23 | 0.29[0.59] | 0.10[0.40] | 0.34[0.31] |
| Anandamide | 1 | 1.01 | 0.01 | 0.83 | 0.57 | 0.44[0.96] | 0.68[0.70] | 0.94[0.92] |
| 2-Arachidonyl glycerol | 1 | 1 | 0 | -0.13 | 0.79 | 0.72[0.75] | 0.29[0.26] | 0.78[0.72] |
| Arachidonic acid | 1 | 0.99 | -0.01 | -0.62 | 0.49 | 0.36[0.43] | 0.11[0.30] | 0.52[0.64] |
| Aminoadipic acid | 1 | 0.98 | -0.02 | -2.05 | 0.10 | 0.36[0.43] | 0.11[0.30] | 0.52[0.64] |
| Homocysteine | 1 | 0.99 | -0.01 | -0.71 | 0.71 | 0.99[0.79] | 0.17[0.63] | 0.56[0.58] |
| Acetoacetic Acid | 1 | 0.99 | -0.01 | -0.57 | 0.53 | 0.22[0.27] | 0.77[0.40] | 0.54[0.92] |
| Isovalerylcarnitine | 1 | 0.99 | -0.01 | -1.35 | **<0.001** | 0.06[0.24] | 0.69[0.68] | 0.66[0.96] |
| 2-methylbutyrylcarnitine | 1 | 0.99 | -0.01 | -1.08 | **0.02** | **0.03**[0.07] | 0.71[0.90] | 0.94[0.89] |
| Propyl carnitine | 1 | 1 | 0 | -0.03 | 0.98 | 0.99[0.92] | 0.11[0.10] | 0.73[0.26] |

Relative change of metabolomics values was calculated as final values divided by baseline values. P values for Model 1 (follow-up adjusted for baseline assessment) are presented, while values in square brackets represent P values derived from Model 2 (further adjusted for age, BMI, sex, and physical activity level). Metabolomics p value have been adjusted for the false discovery rate (Benjamini & Hochber).
